# Supplementary figures and images for: Mashes to Mashes, Crust to Crust. Presenting a novel microstructural marker for malting in the archaeological record
Source: PLoS One. 2020 May 7;15(5):e0231696. doi: 10.1371/journal.pone.0231696 (PMC7205394; doi:10.1371/journal.pone.0231696)

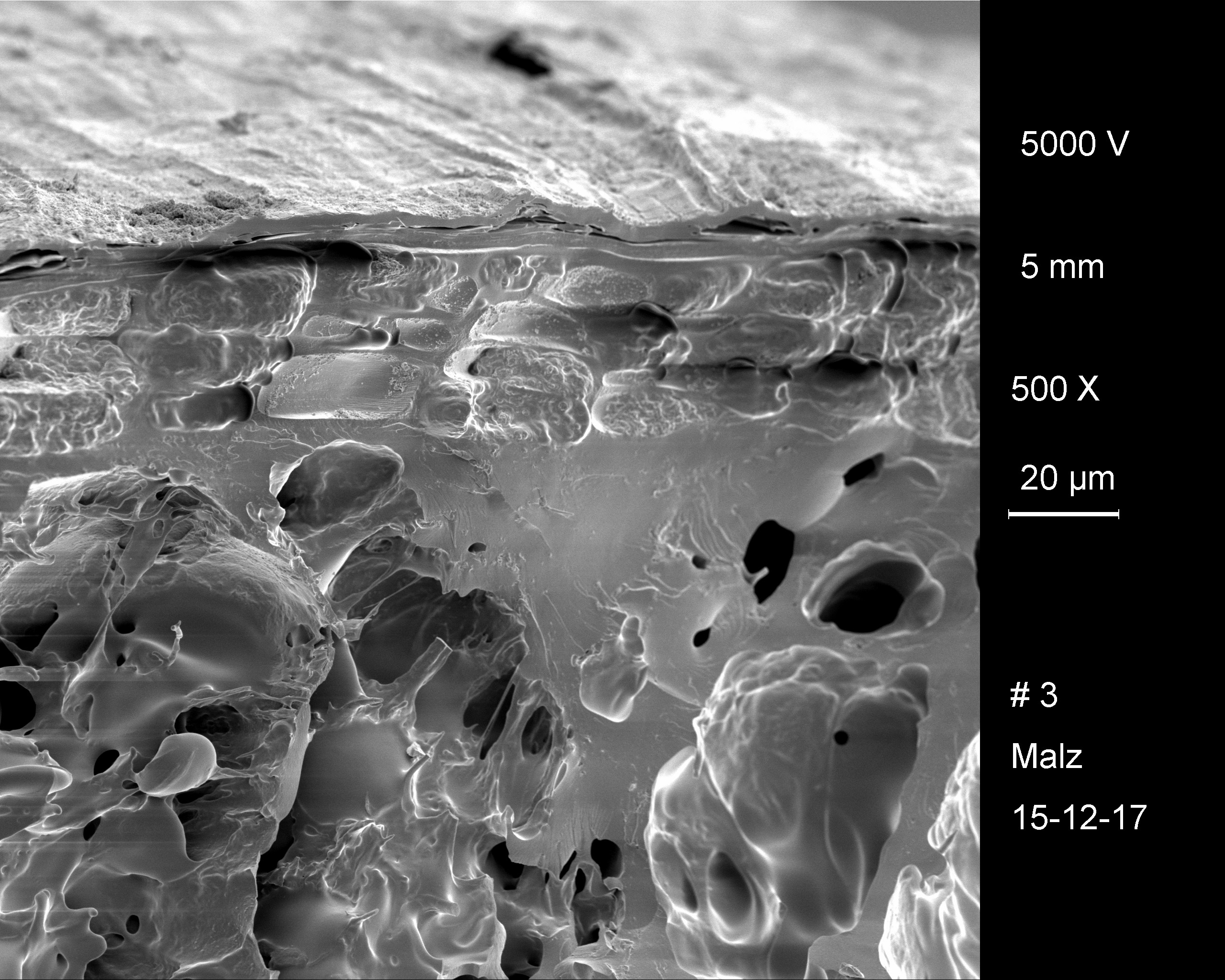

Supplement: S1 Archive — (ZIP) [file pone.0231696.s003.zip › HOVUS_M1_C_01.jpg]

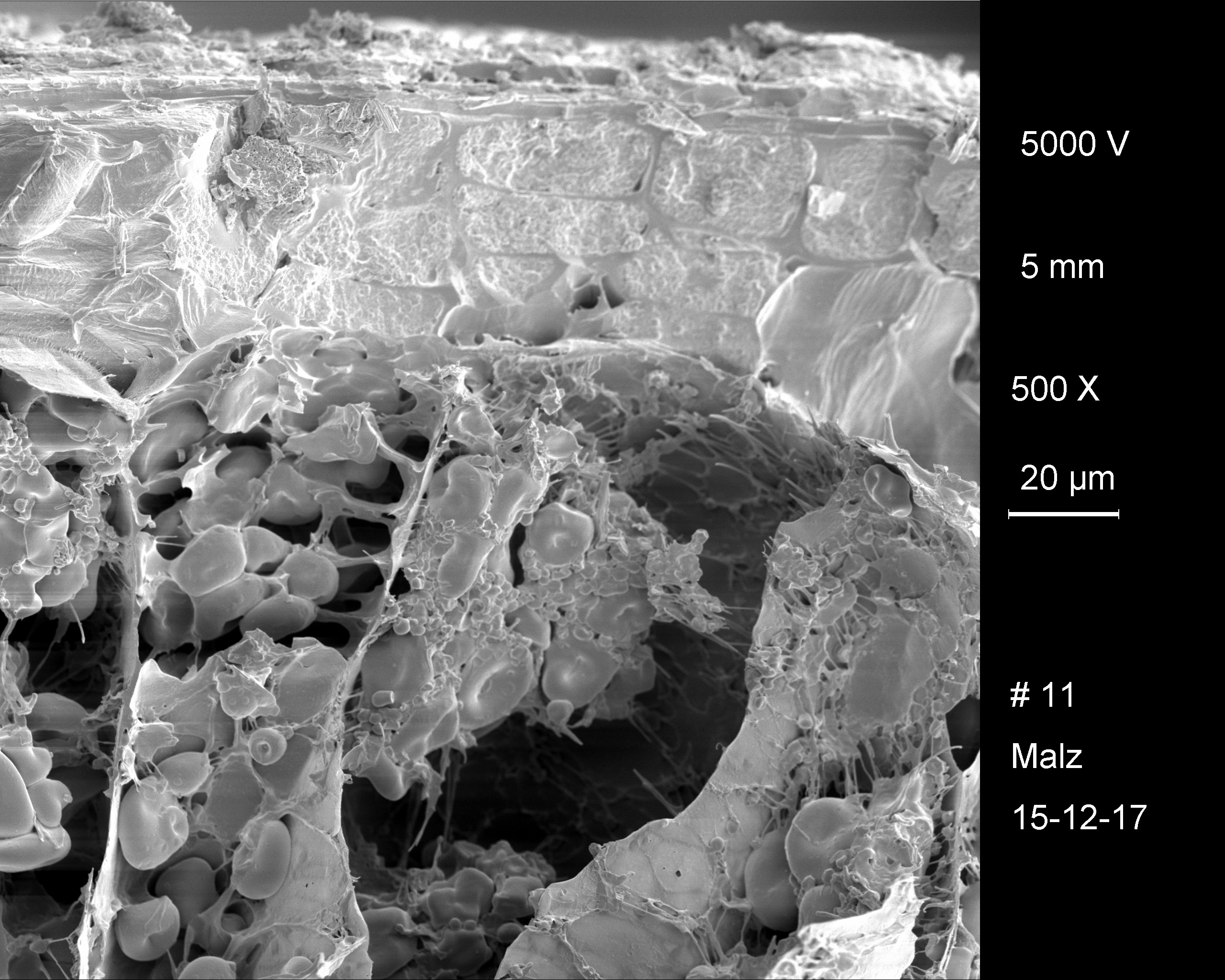

Supplement: S1 Archive — (ZIP) [file pone.0231696.s003.zip › HOVUS_M1_C_02.jpg]

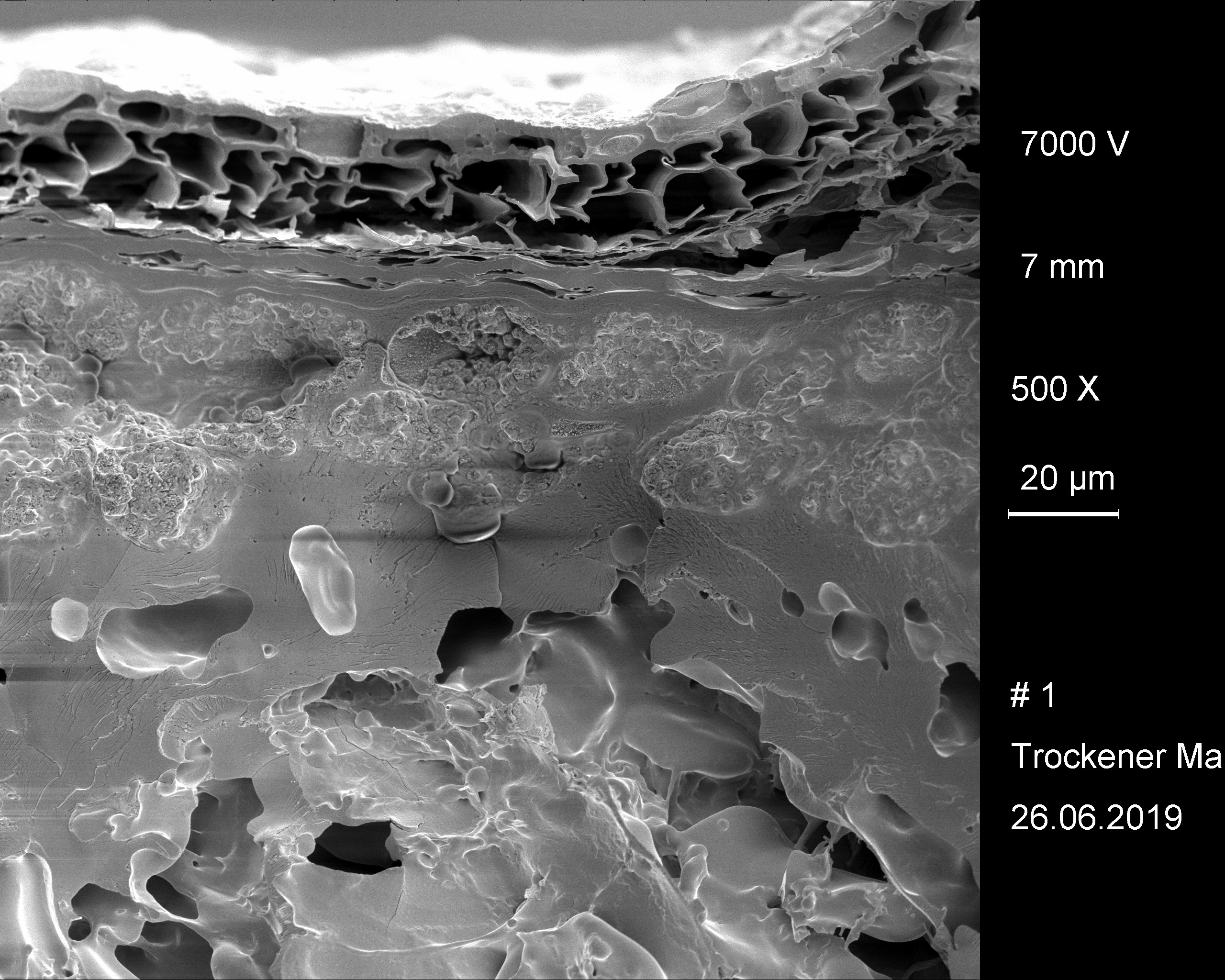

Supplement: S1 Archive — (ZIP) [file pone.0231696.s003.zip › HOVUS_M1_C_03.jpg]

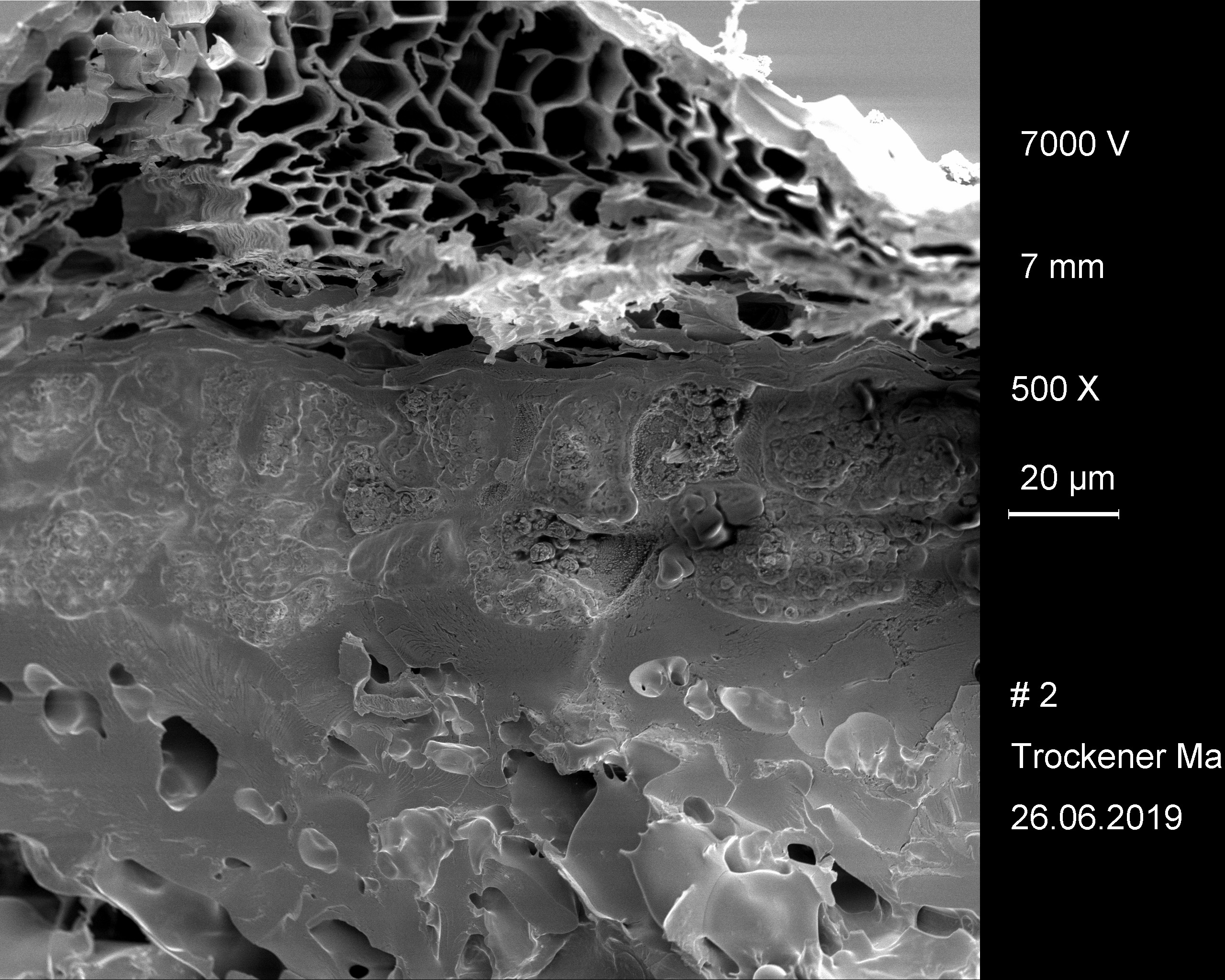

Supplement: S1 Archive — (ZIP) [file pone.0231696.s003.zip › HOVUS_M1_C_04.jpg]

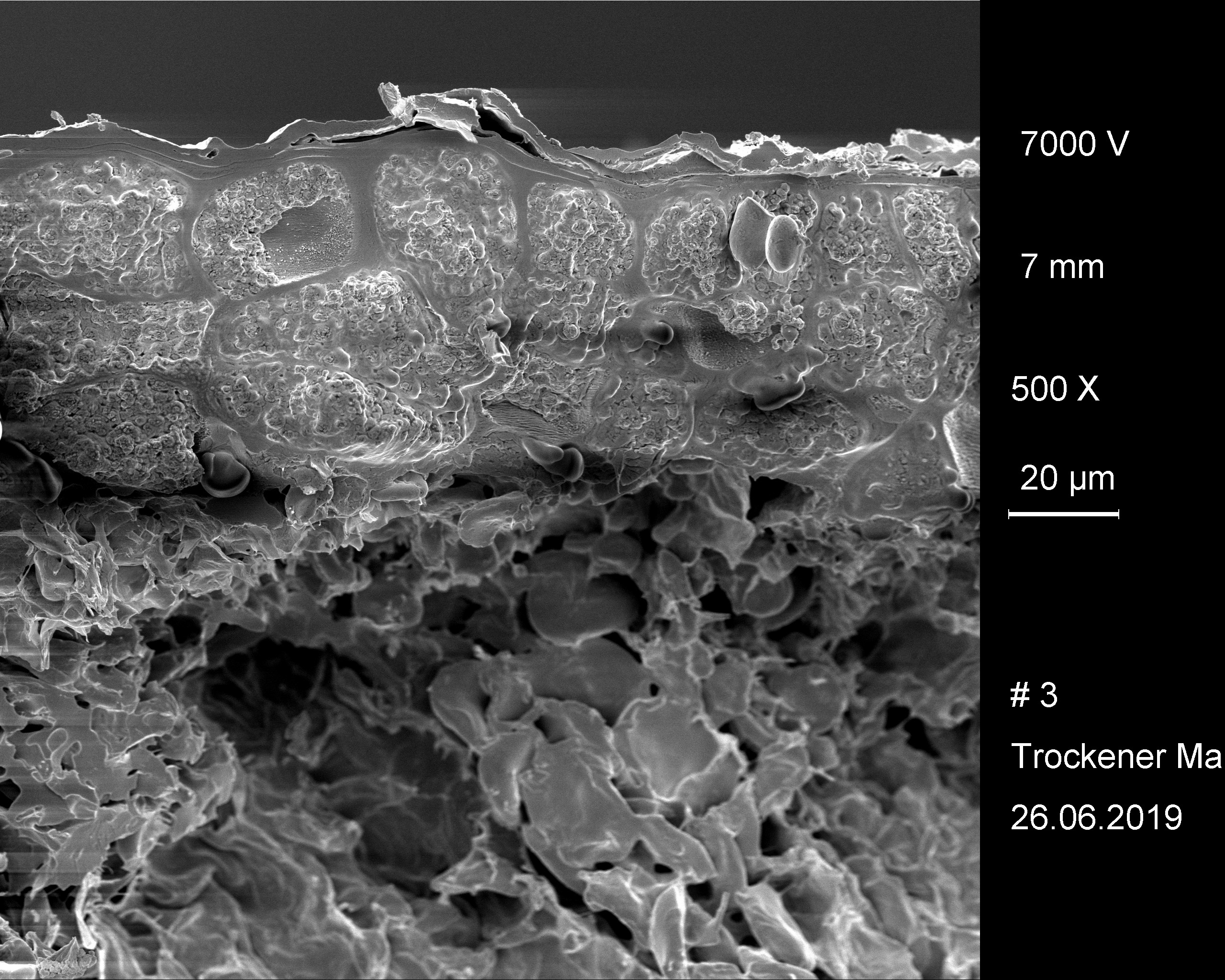

Supplement: S1 Archive — (ZIP) [file pone.0231696.s003.zip › HOVUS_M1_C_05.jpg]

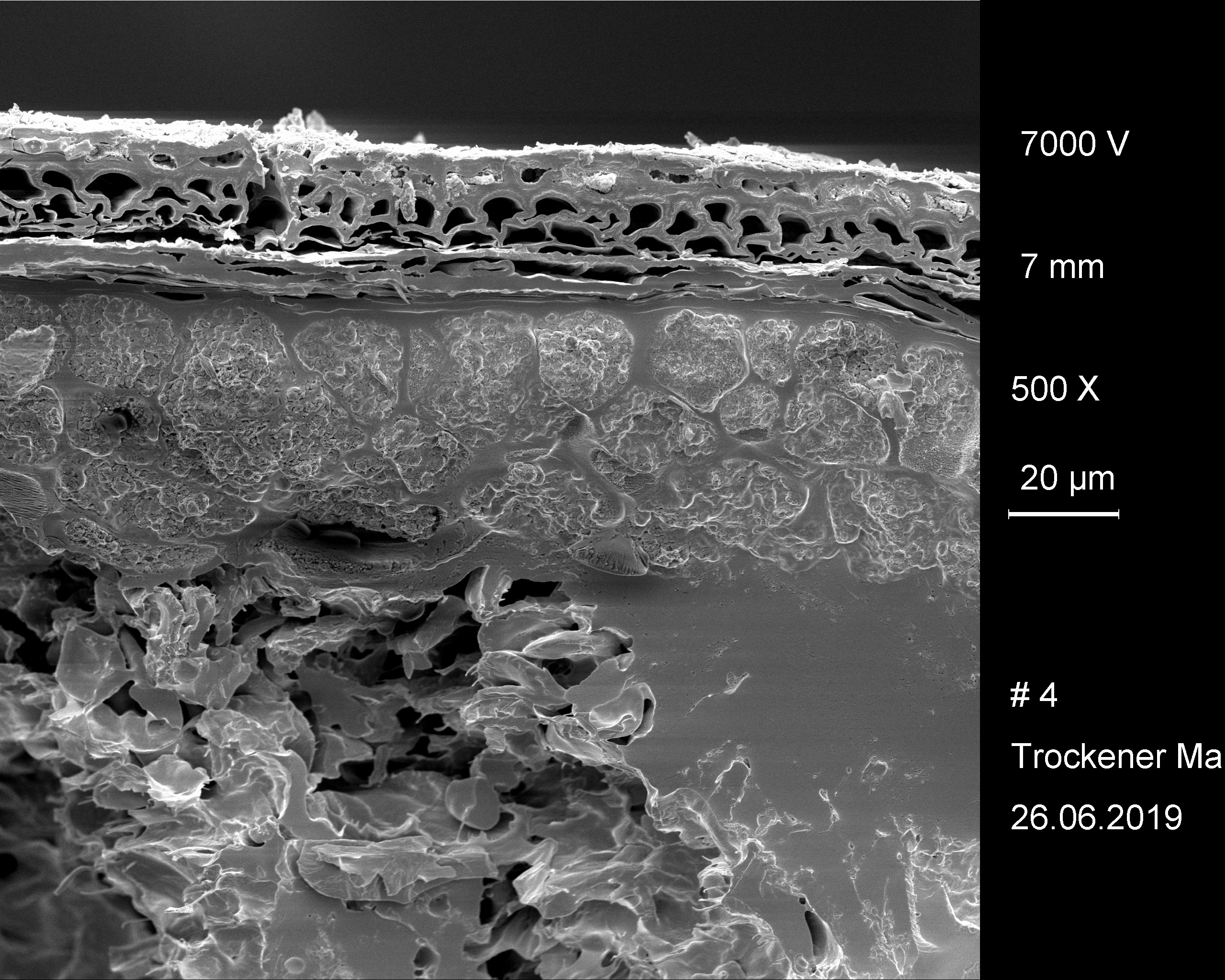

Supplement: S1 Archive — (ZIP) [file pone.0231696.s003.zip › HOVUS_M1_C_06.jpg]

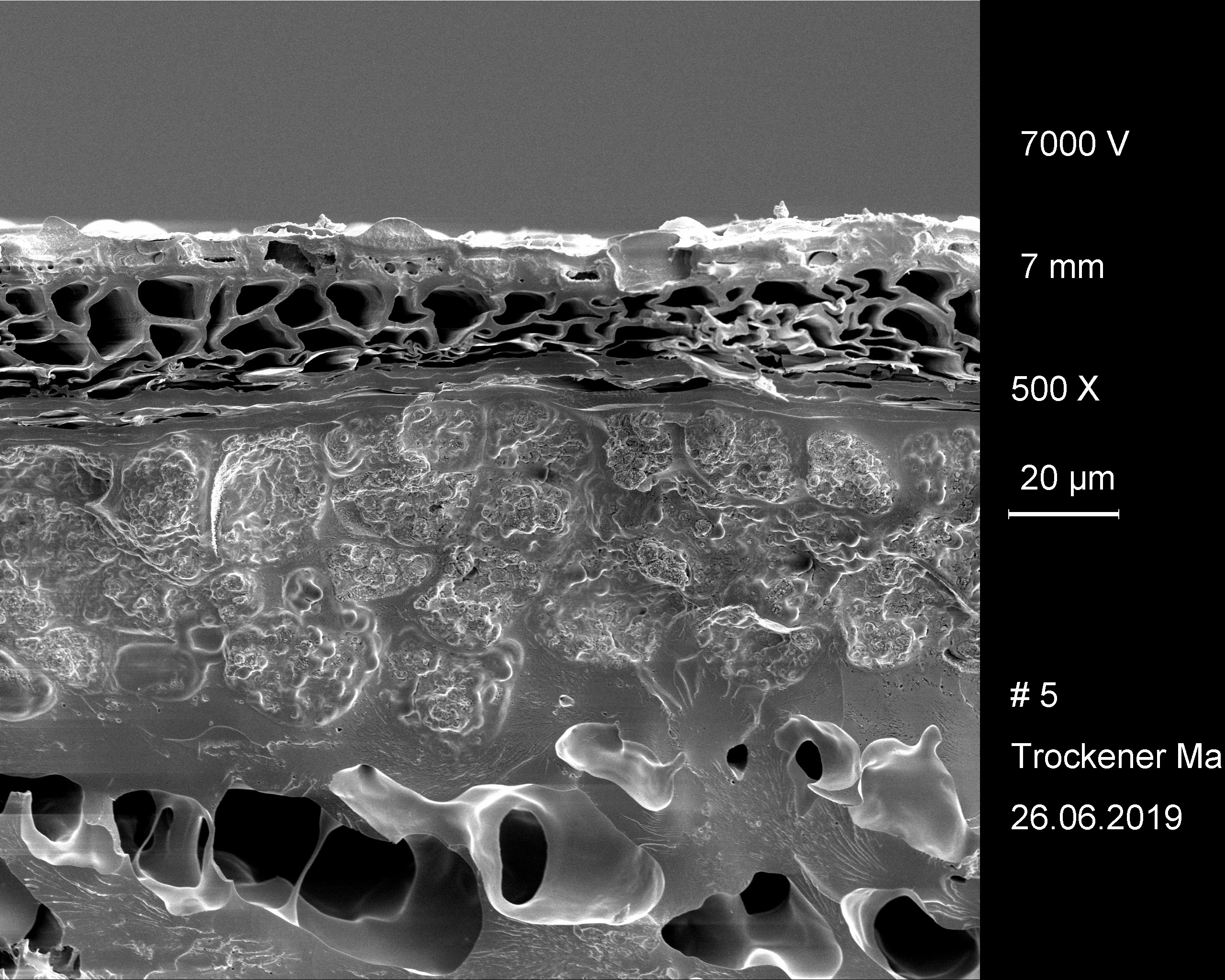

Supplement: S1 Archive — (ZIP) [file pone.0231696.s003.zip › HOVUS_M1_C_07.jpg]

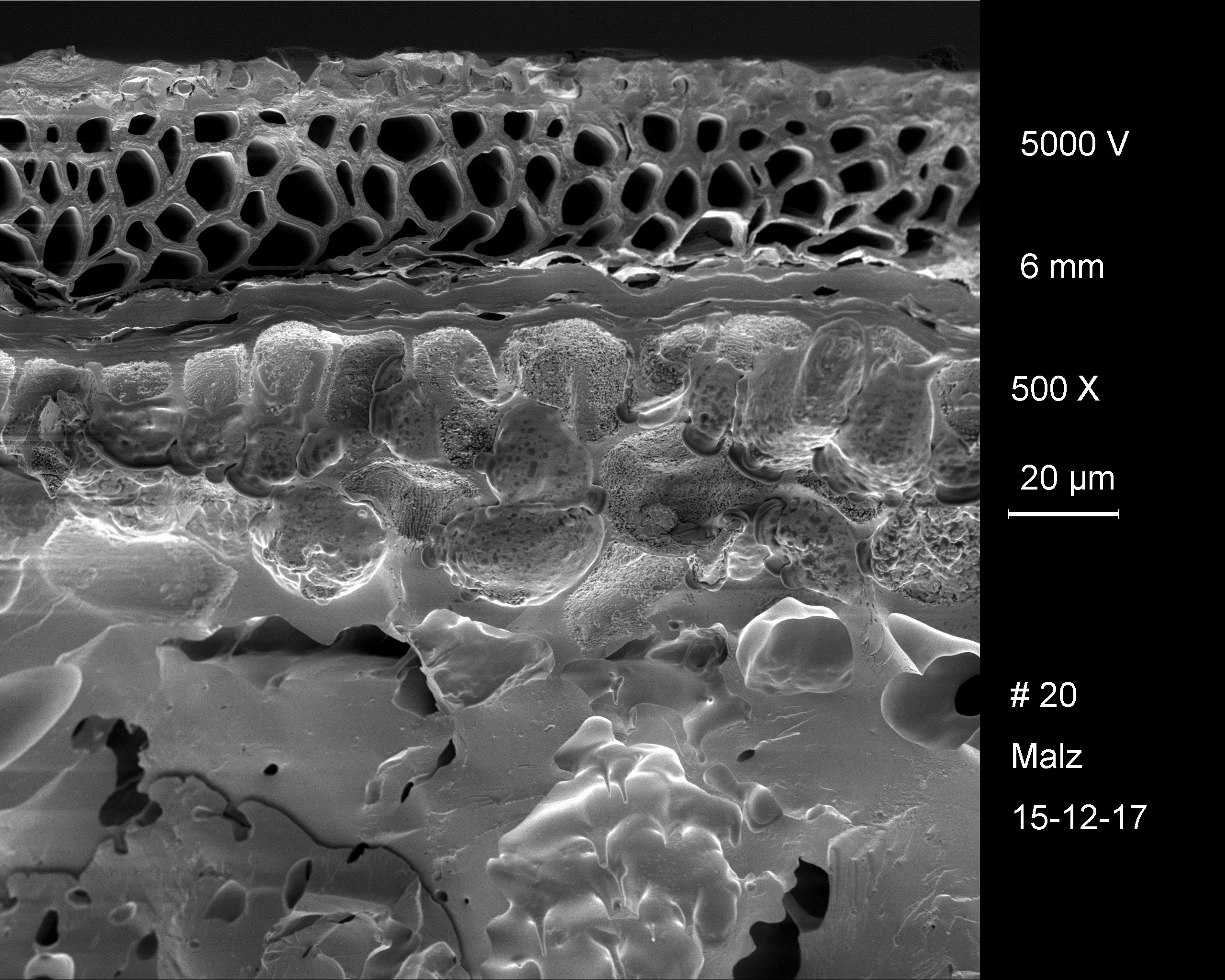

Supplement: S1 Archive — (ZIP) [file pone.0231696.s003.zip › HOVUS_M2_C_01.jpg]

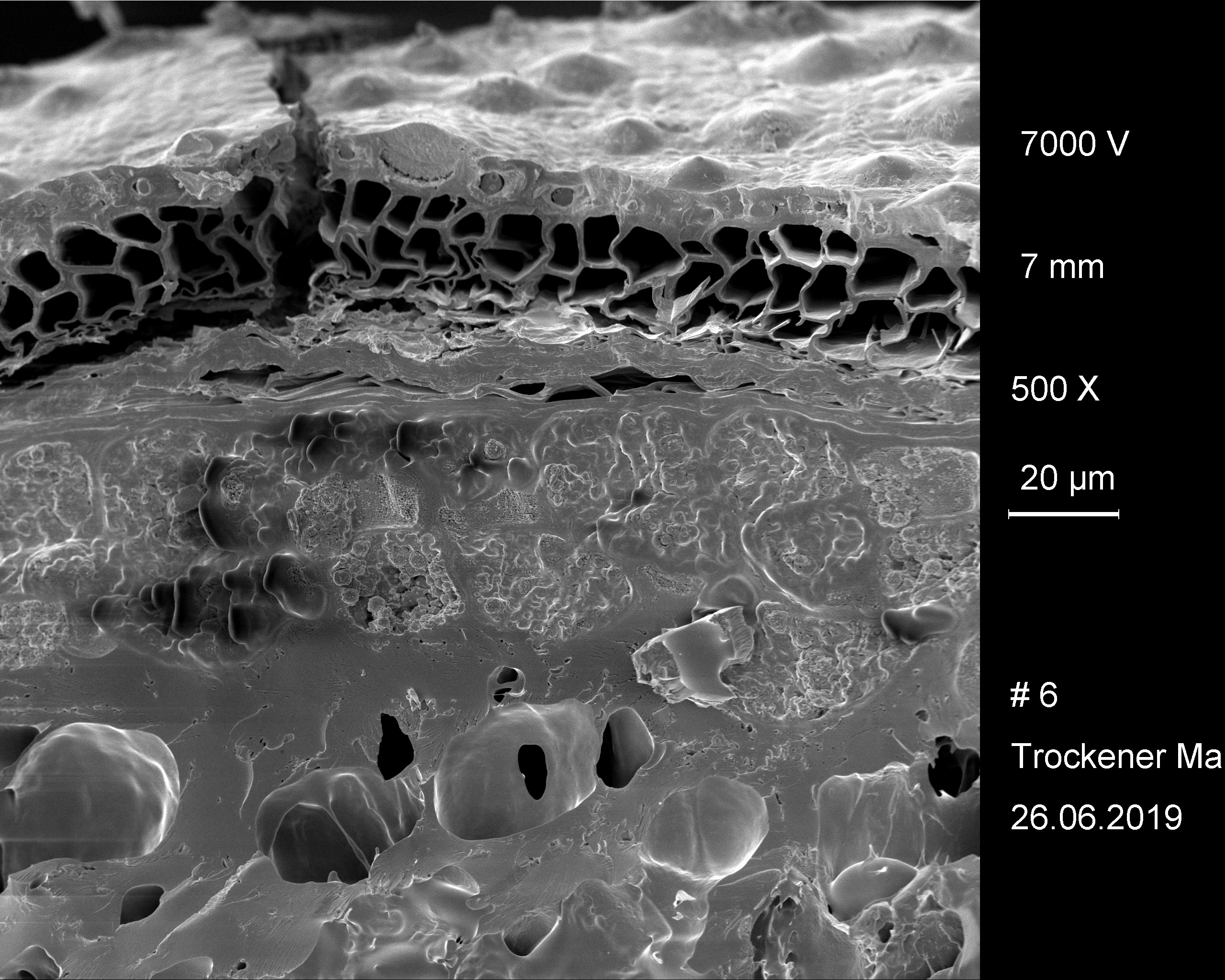

Supplement: S1 Archive — (ZIP) [file pone.0231696.s003.zip › HOVUS_M2_C_02.jpg]

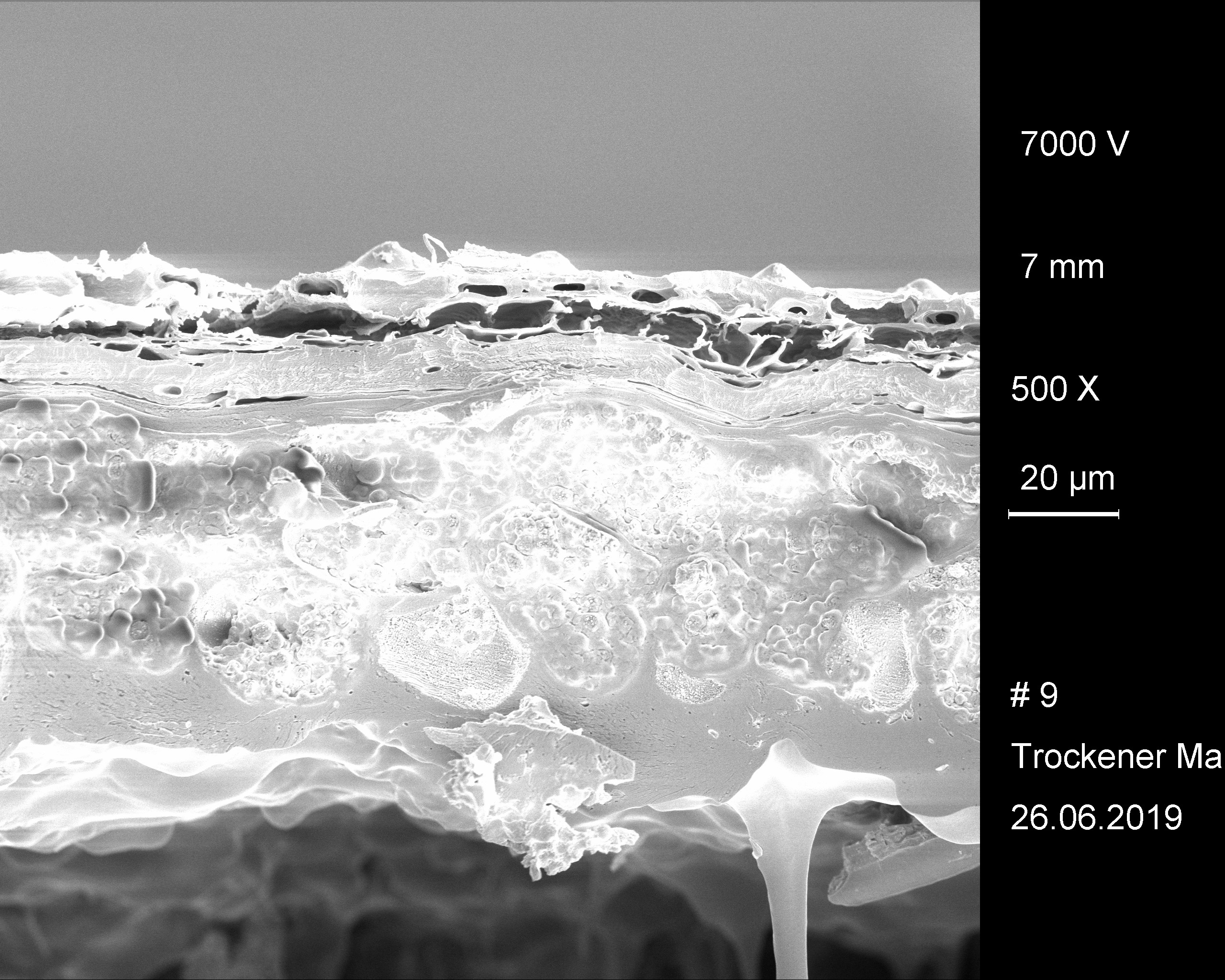

Supplement: S1 Archive — (ZIP) [file pone.0231696.s003.zip › HOVUS_M2_C_03.jpg]

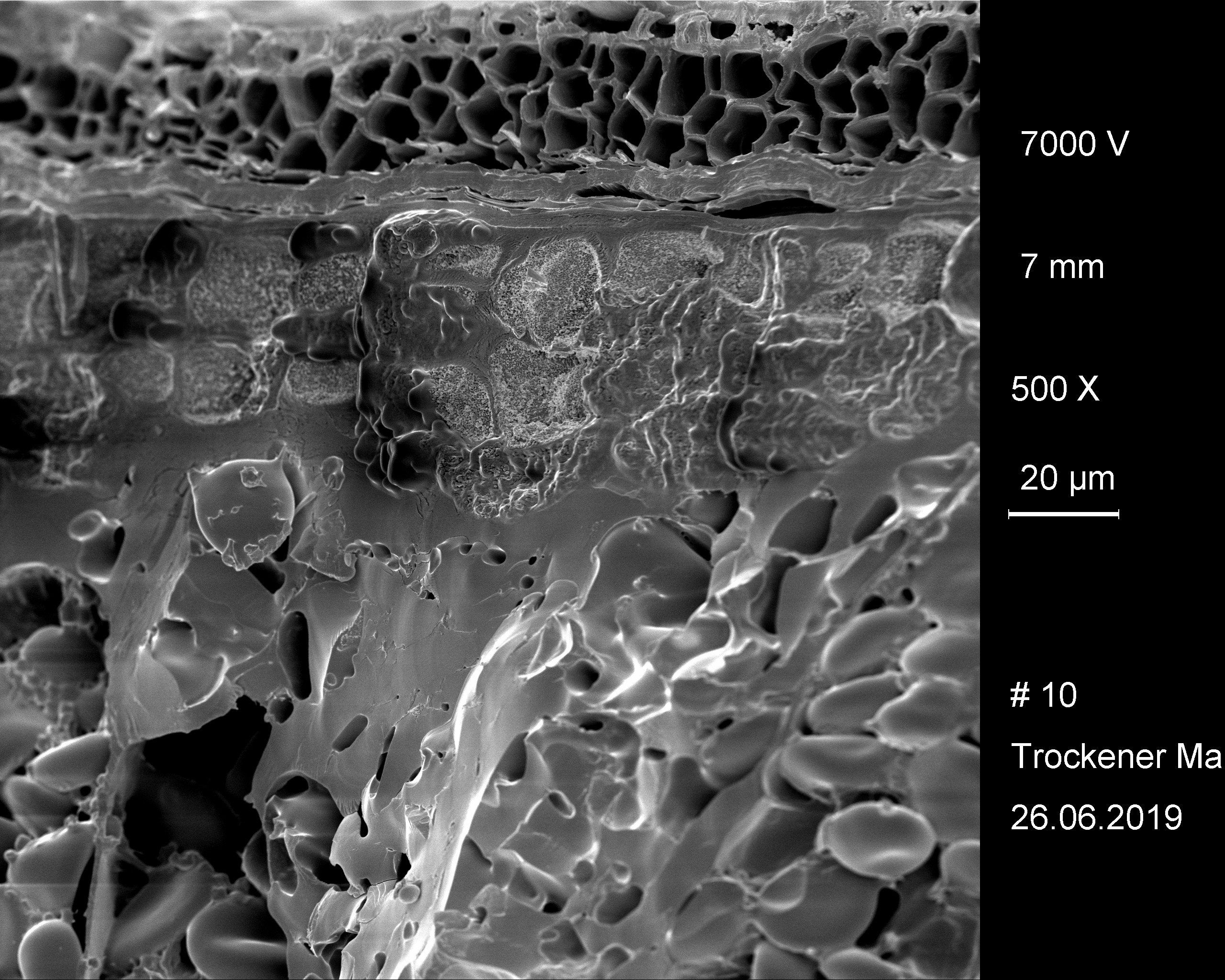

Supplement: S1 Archive — (ZIP) [file pone.0231696.s003.zip › HOVUS_M2_C_04.jpg]

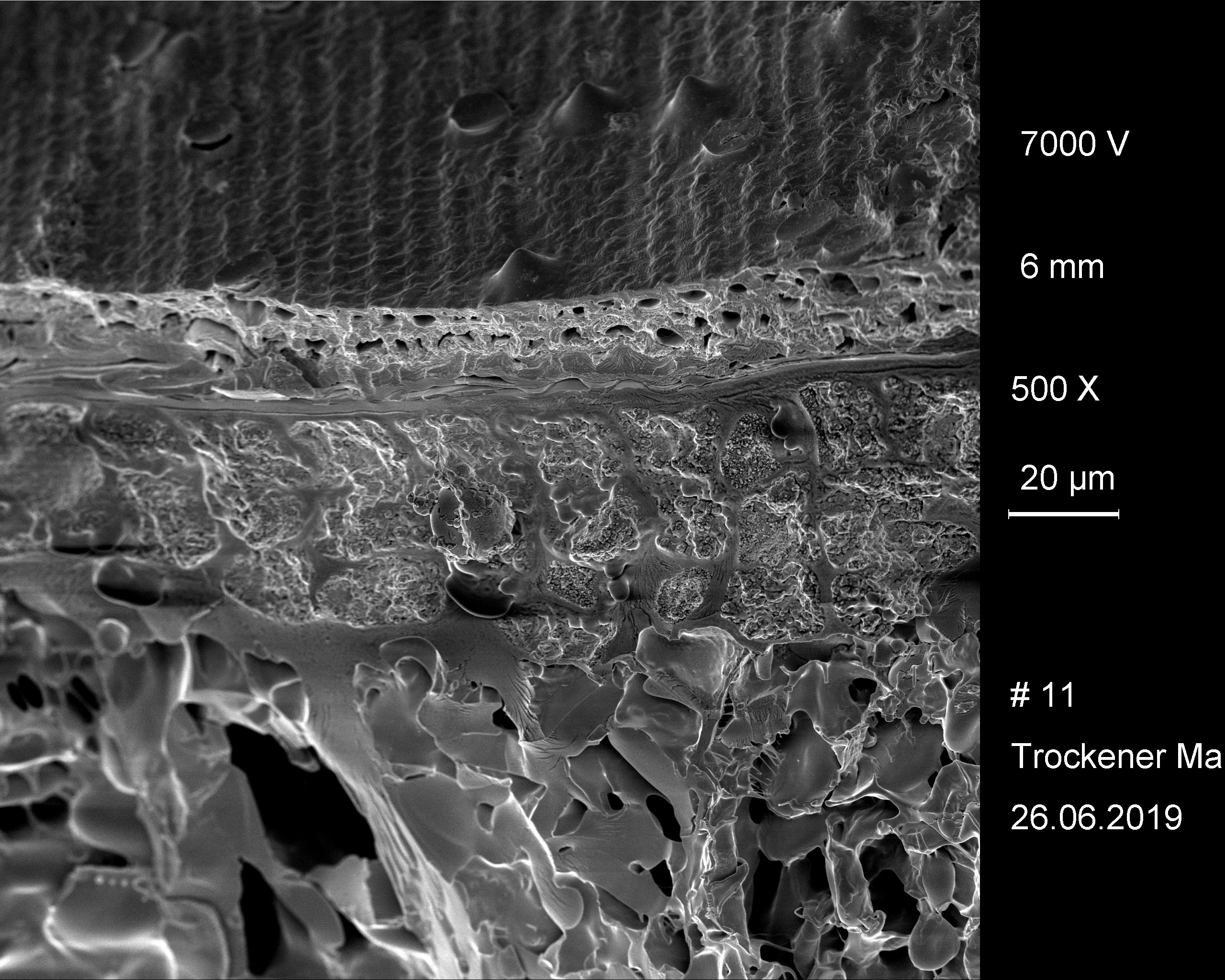

Supplement: S1 Archive — (ZIP) [file pone.0231696.s003.zip › HOVUS_M2_C_05.jpg]

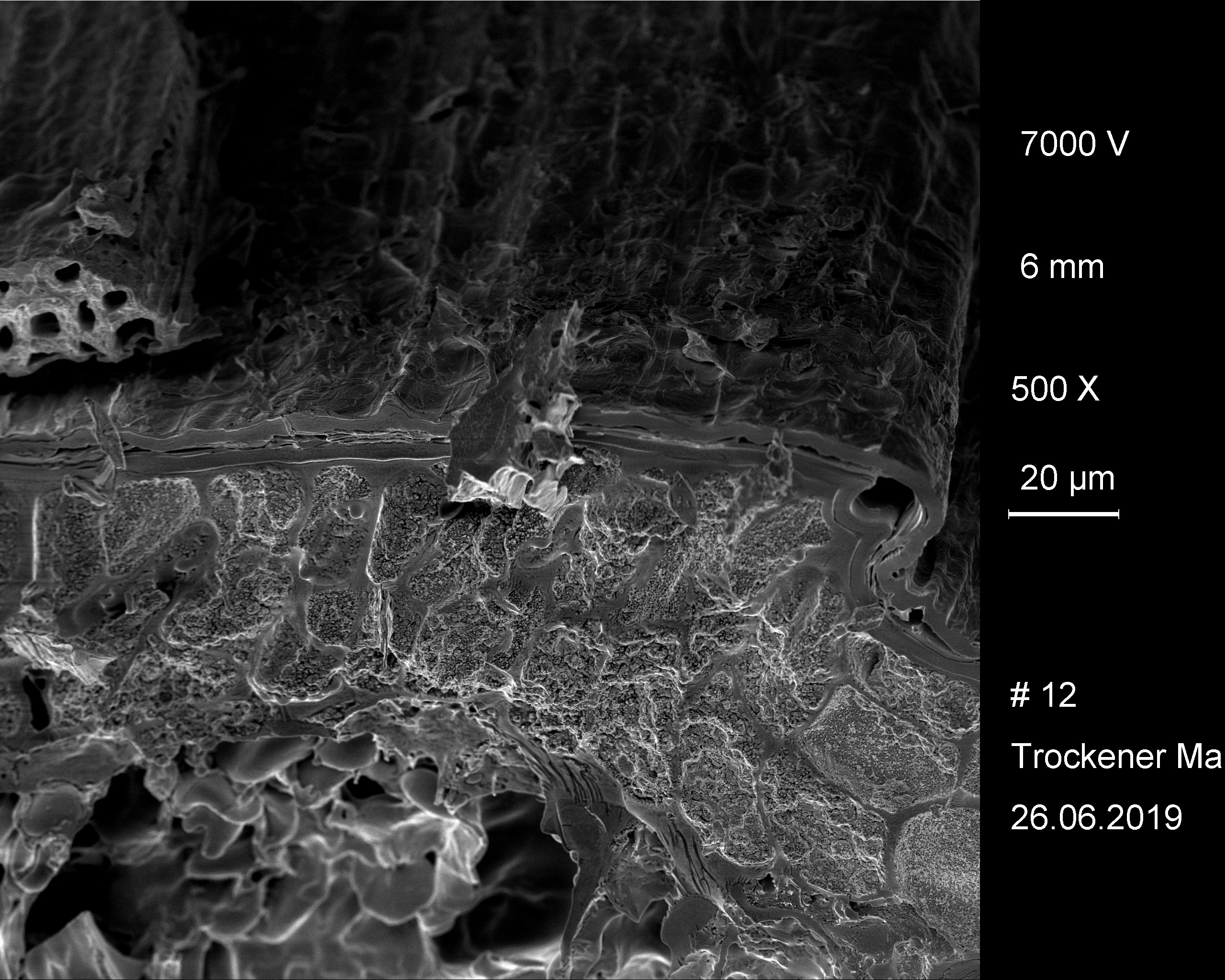

Supplement: S1 Archive — (ZIP) [file pone.0231696.s003.zip › HOVUS_M2_C_06.jpg]

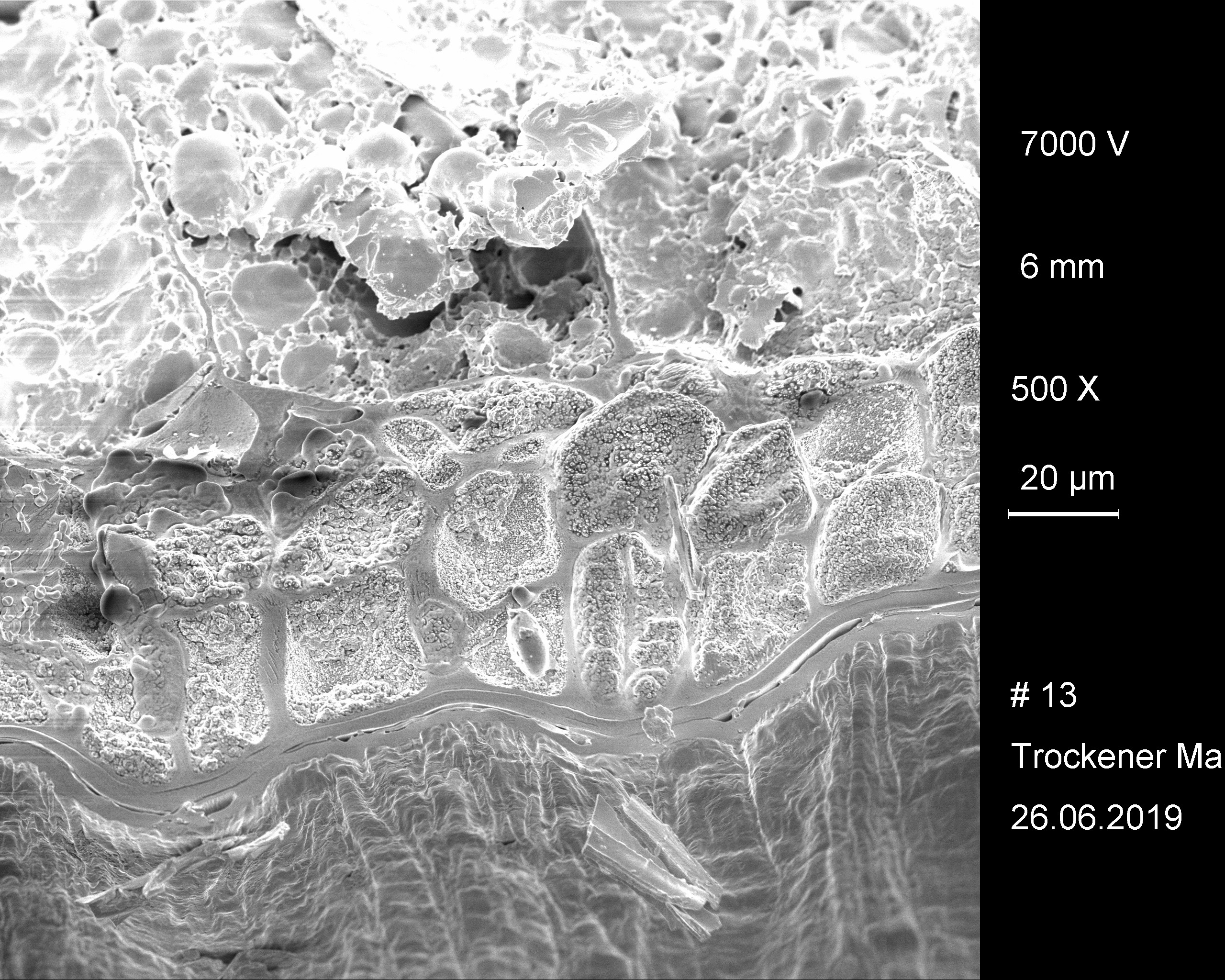

Supplement: S1 Archive — (ZIP) [file pone.0231696.s003.zip › HOVUS_M2_C_07.jpg]

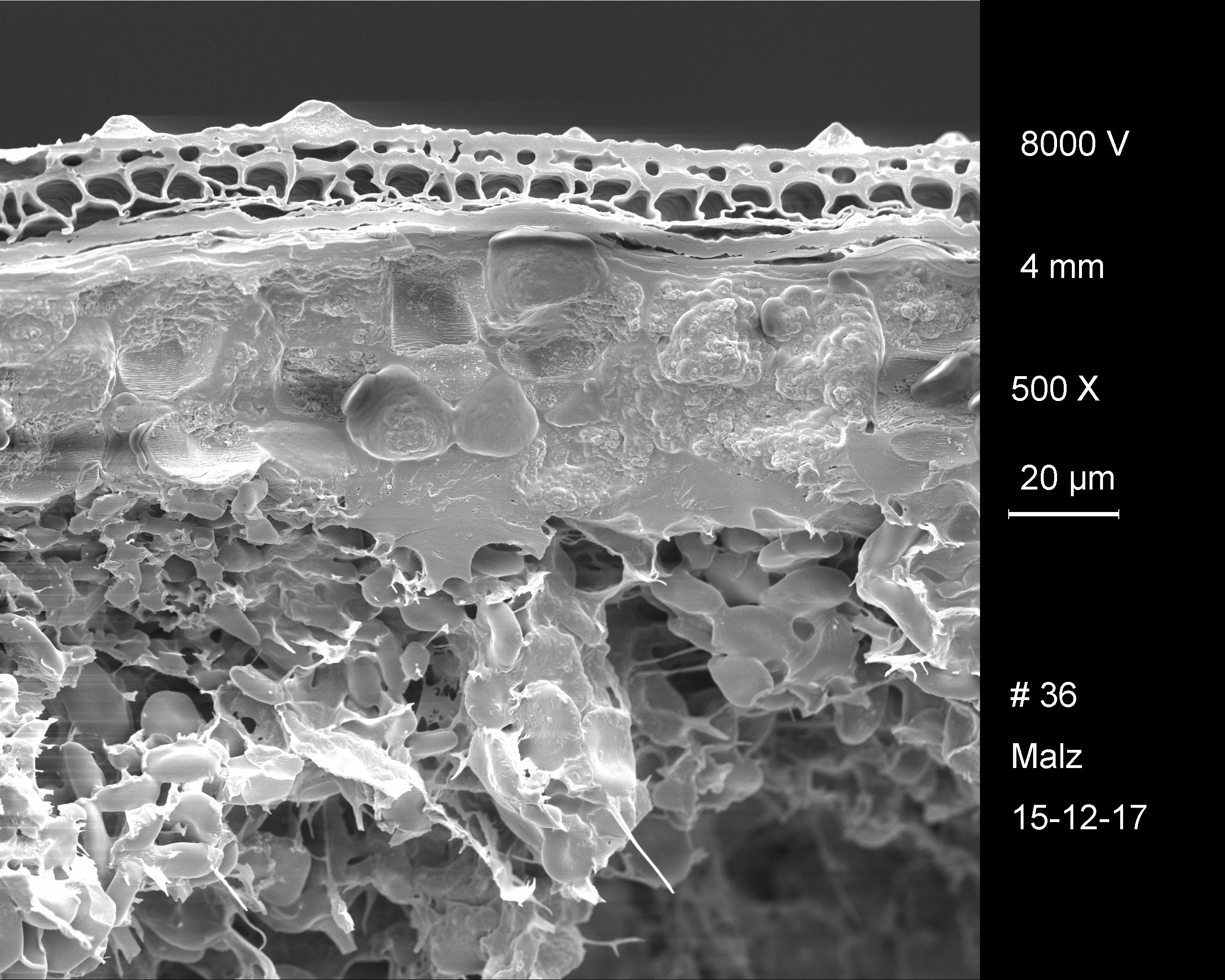

Supplement: S1 Archive — (ZIP) [file pone.0231696.s003.zip › HOVUS_M3_C_01.jpg]

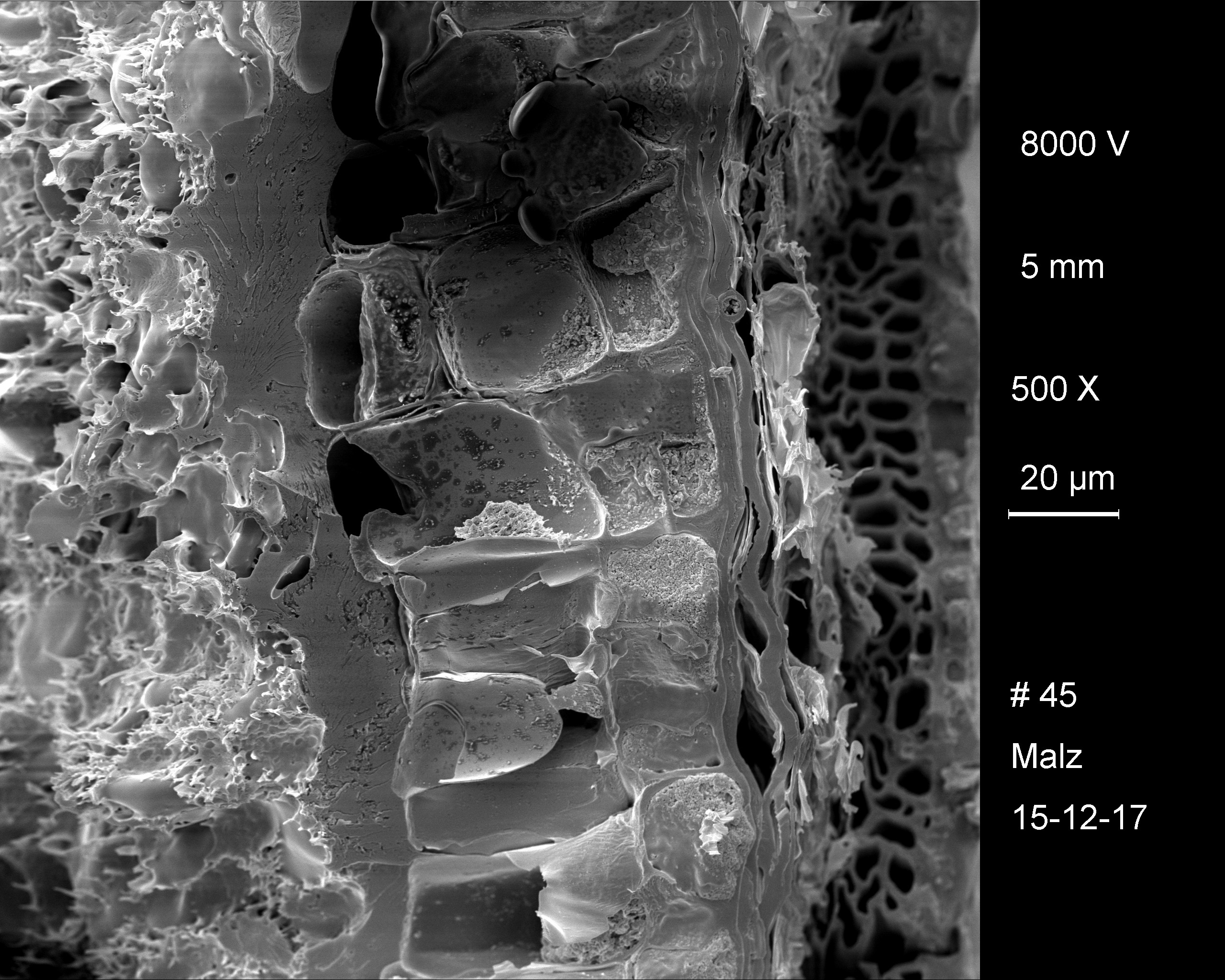

Supplement: S1 Archive — (ZIP) [file pone.0231696.s003.zip › HOVUS_M3_C_02.jpg]

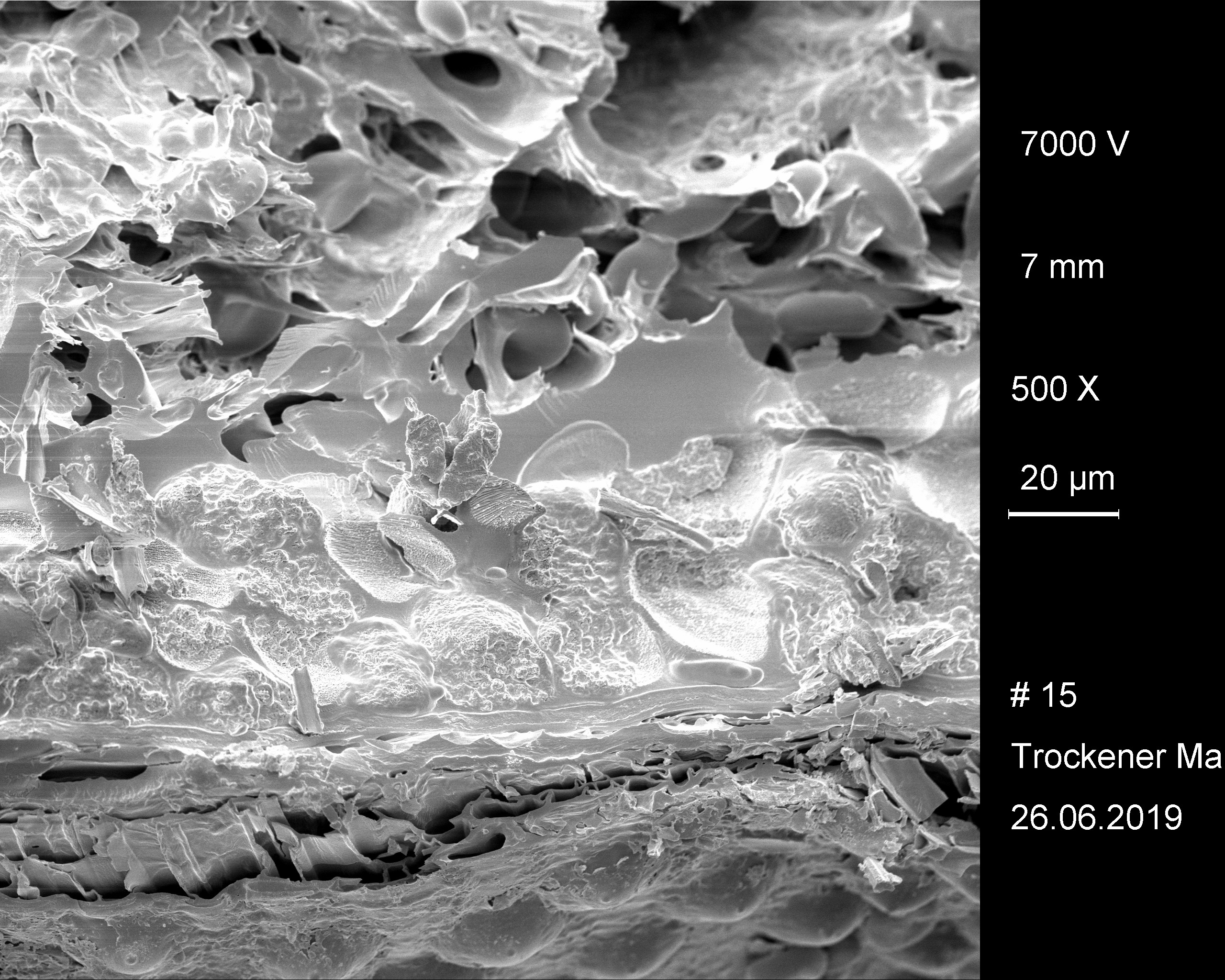

Supplement: S1 Archive — (ZIP) [file pone.0231696.s003.zip › HOVUS_M3_C_03.jpg]

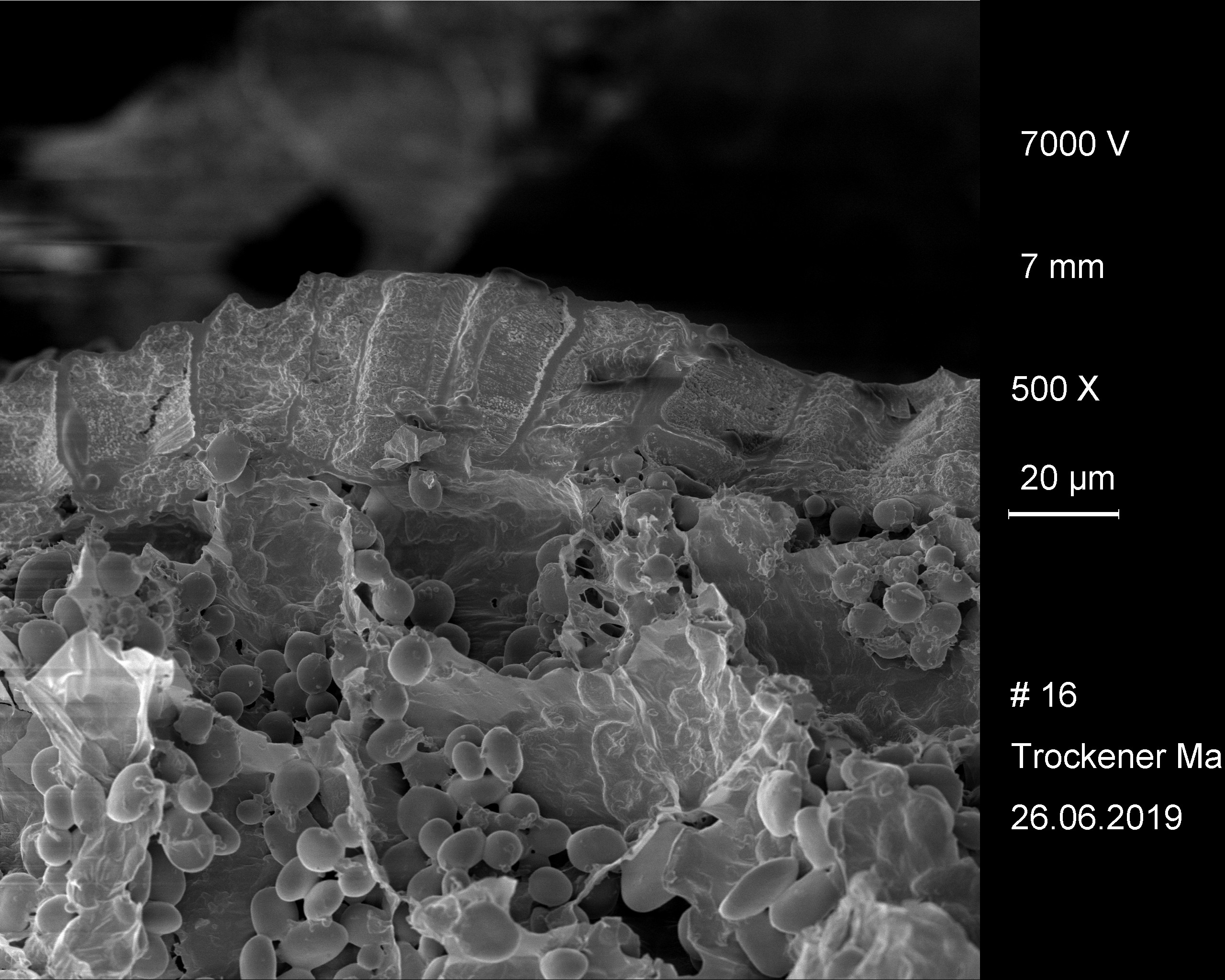

Supplement: S1 Archive — (ZIP) [file pone.0231696.s003.zip › HOVUS_M3_C_04.jpg]

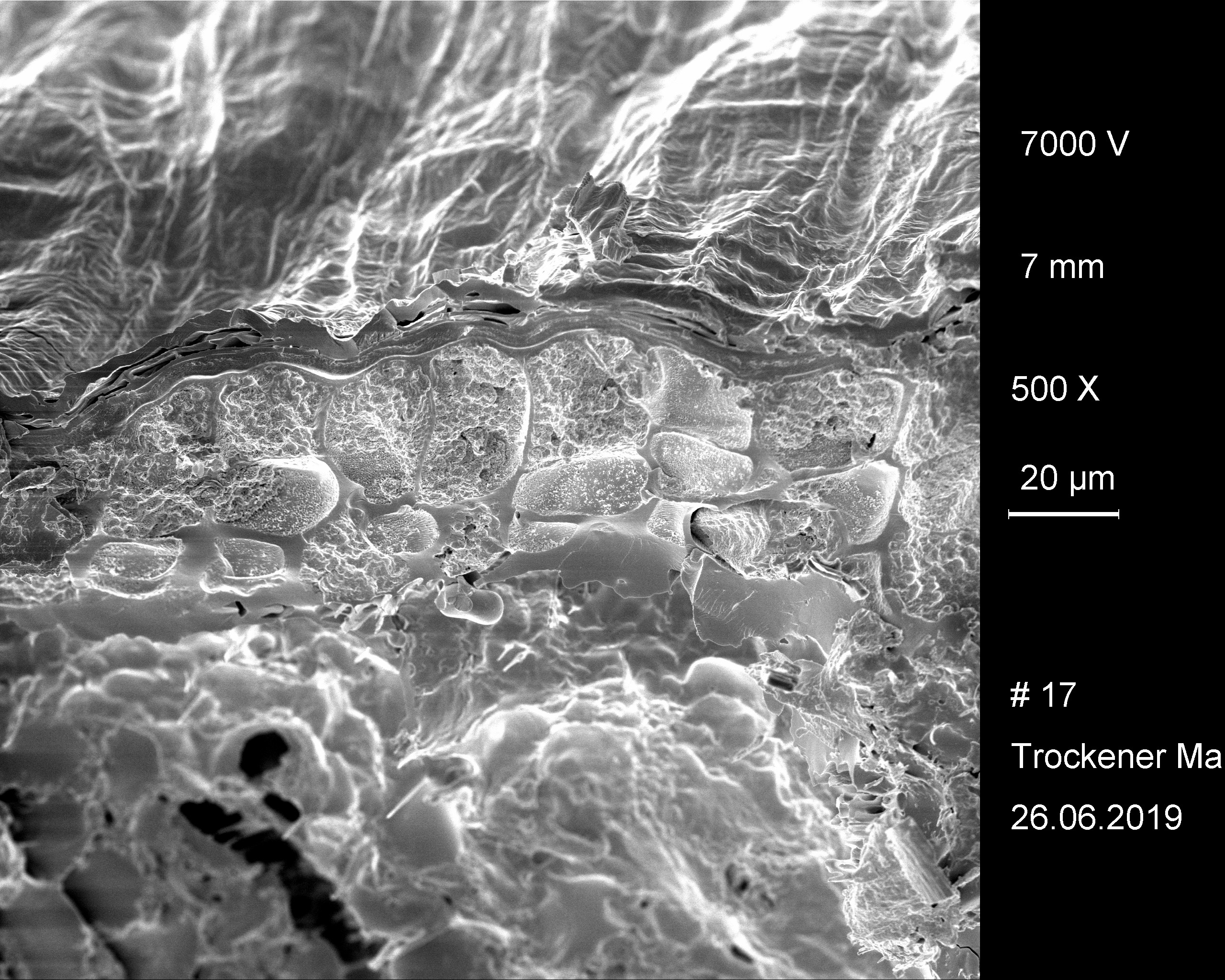

Supplement: S1 Archive — (ZIP) [file pone.0231696.s003.zip › HOVUS_M3_C_05.jpg]

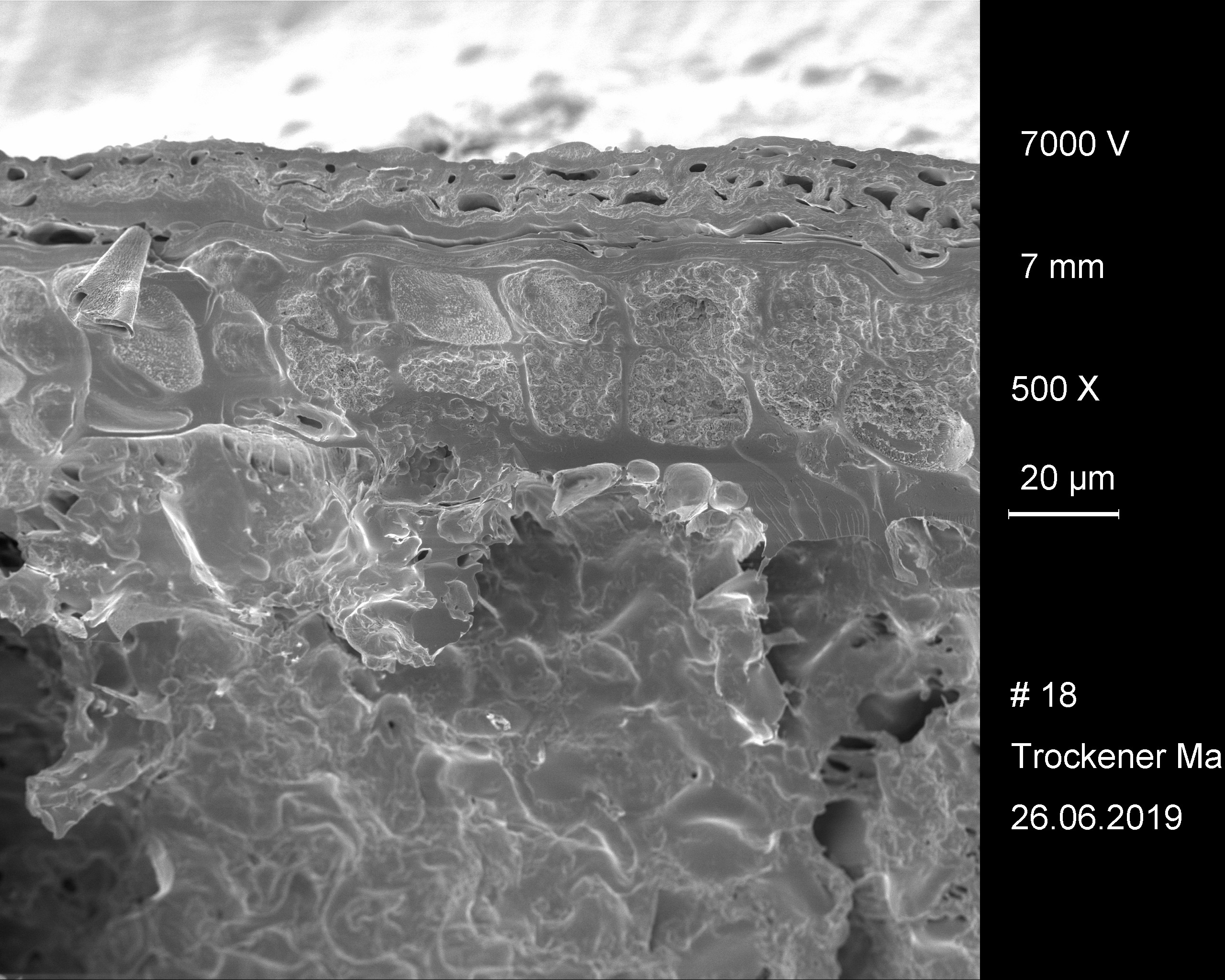

Supplement: S1 Archive — (ZIP) [file pone.0231696.s003.zip › HOVUS_M3_C_06.jpg]

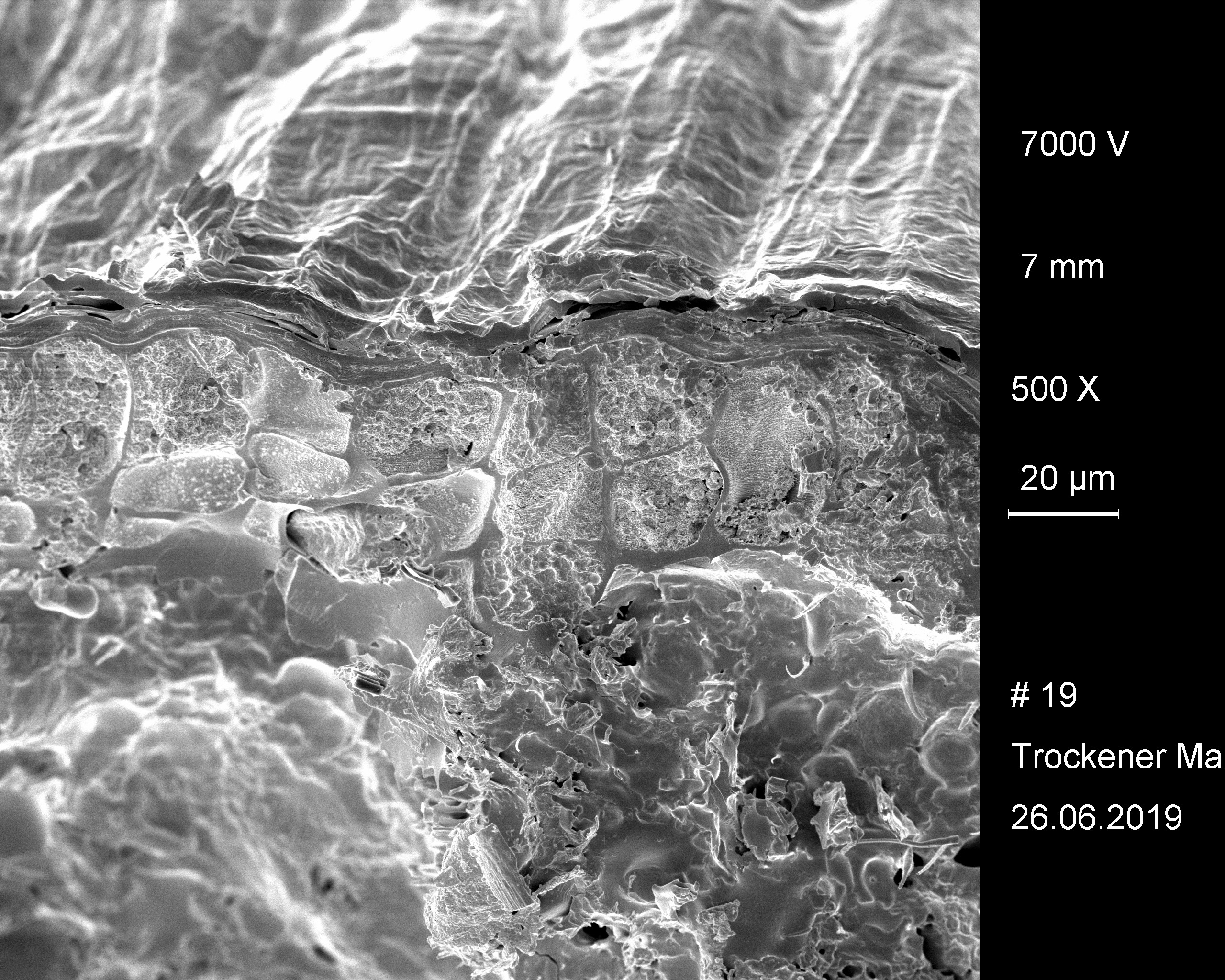

Supplement: S1 Archive — (ZIP) [file pone.0231696.s003.zip › HOVUS_M3_C_07.jpg]

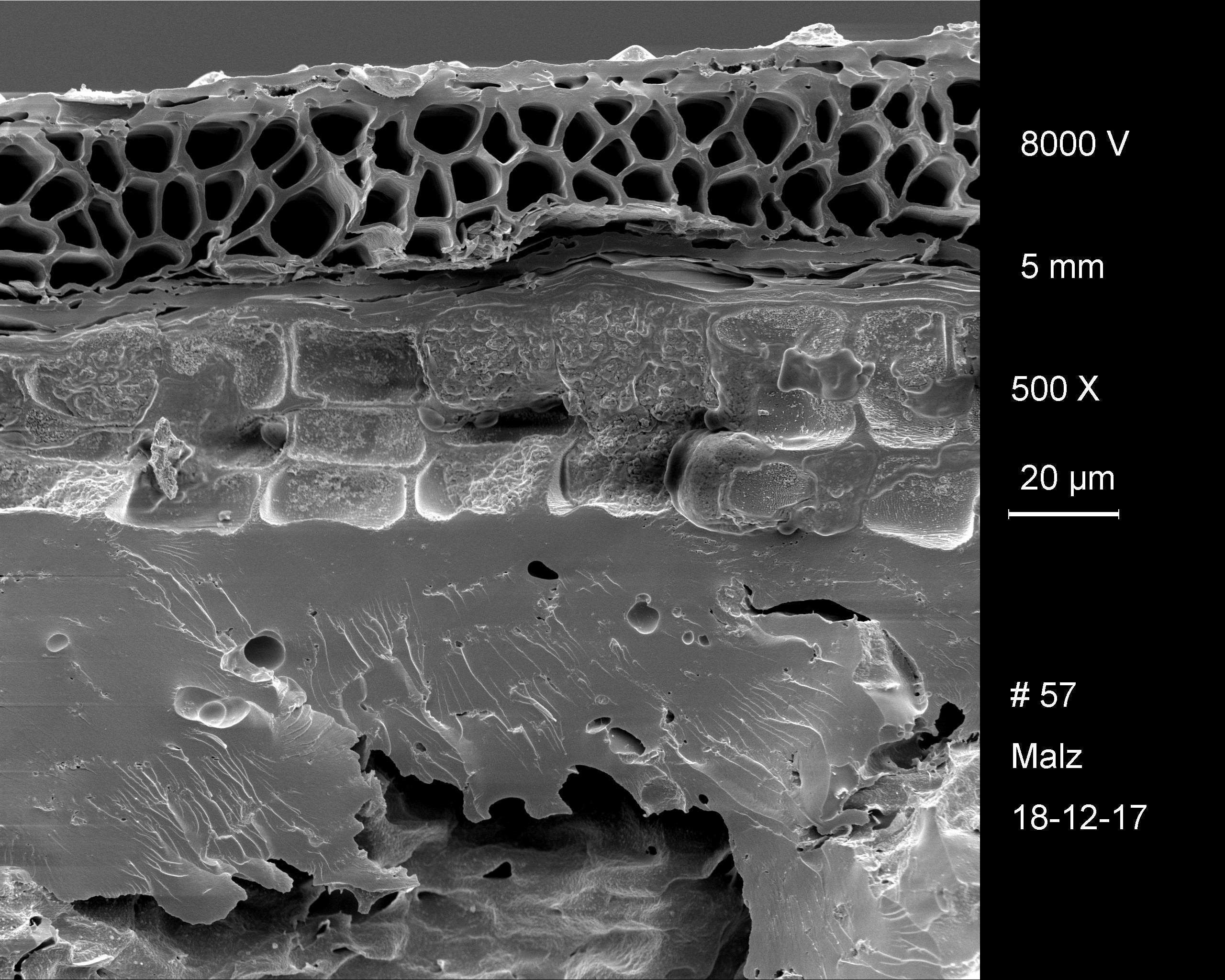

Supplement: S1 Archive — (ZIP) [file pone.0231696.s003.zip › HOVUS_M4_C_01.jpg]

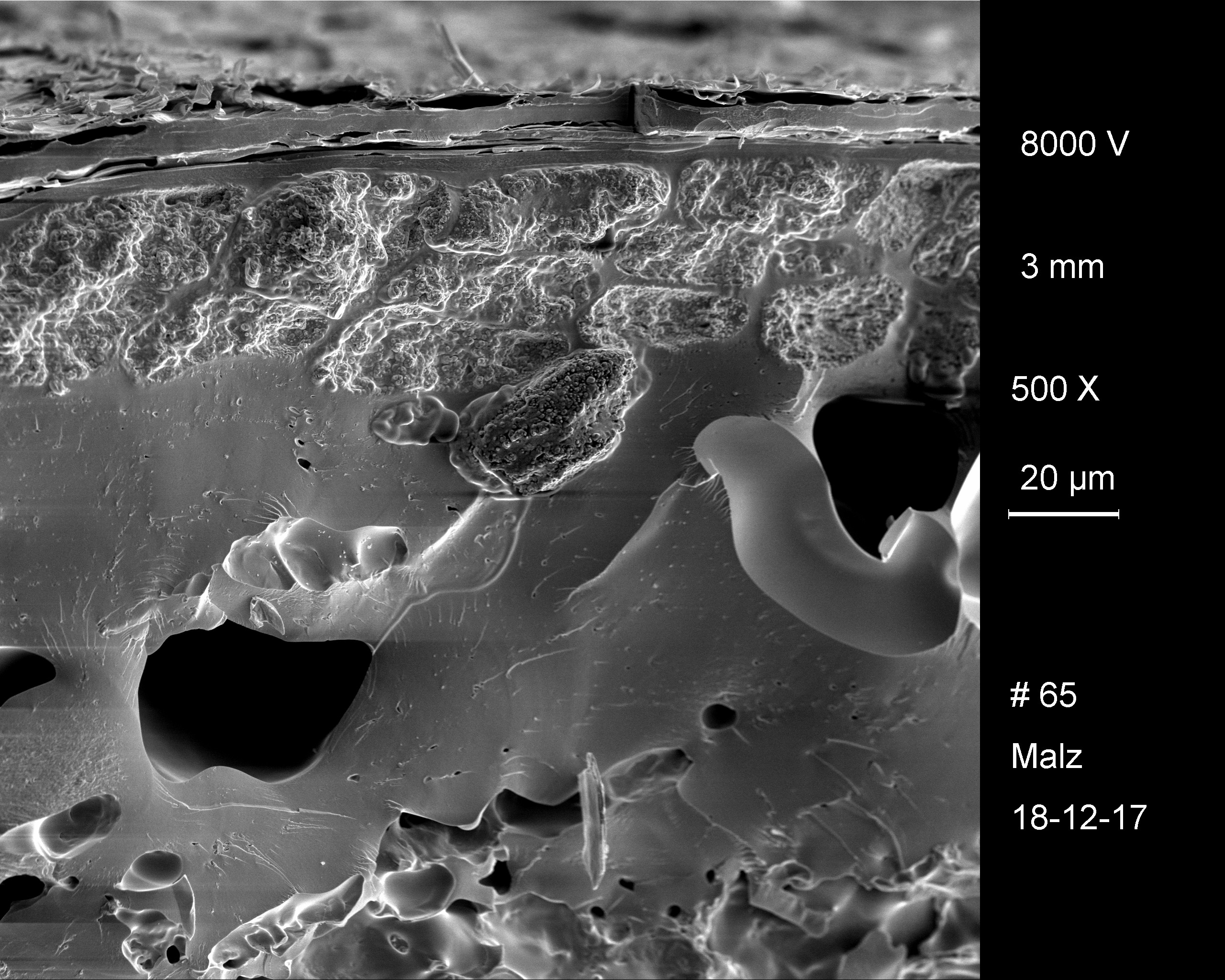

Supplement: S1 Archive — (ZIP) [file pone.0231696.s003.zip › HOVUS_M4_C_02.jpg]

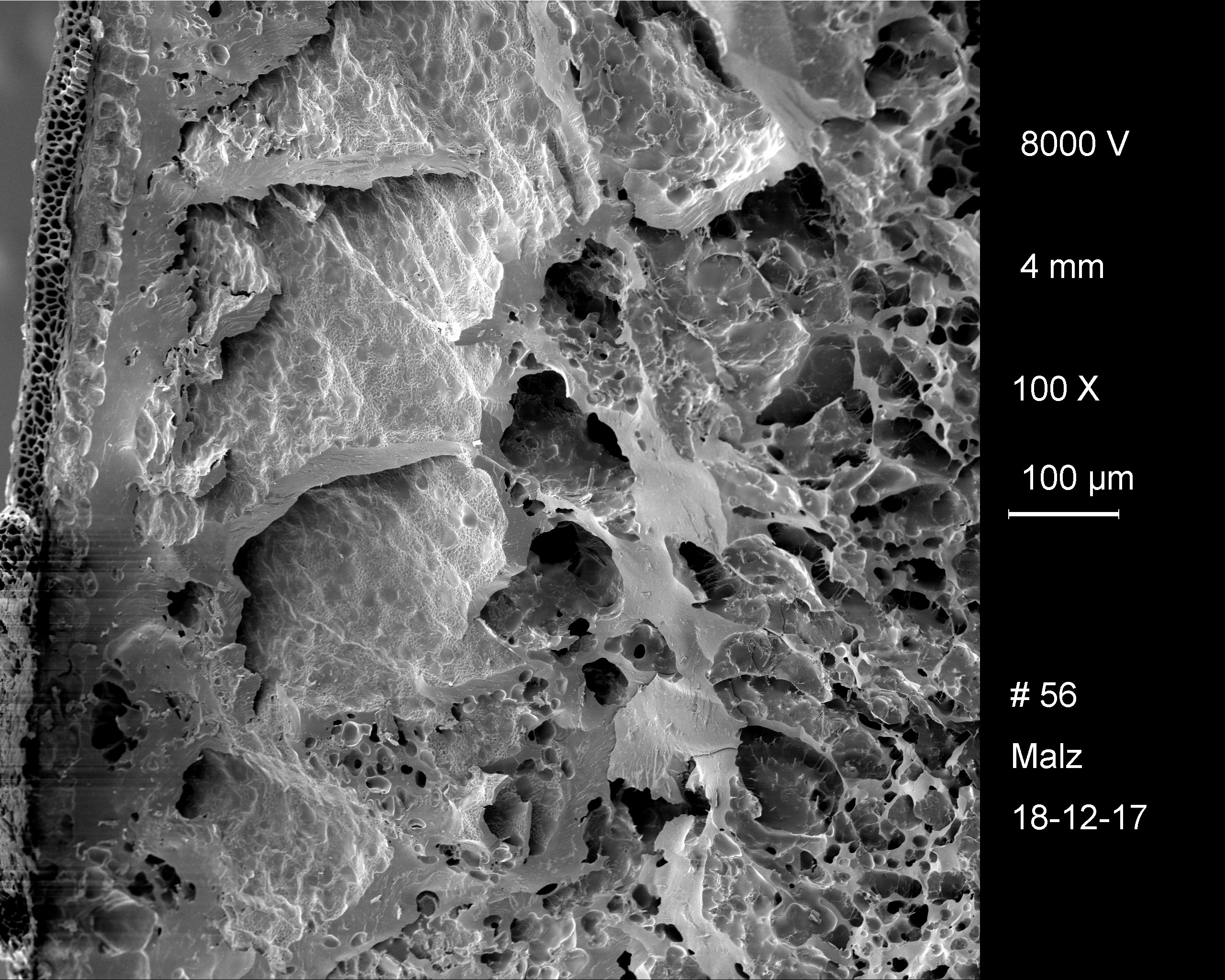

Supplement: S1 Archive — (ZIP) [file pone.0231696.s003.zip › HOVUS_M4_C_03.jpg]

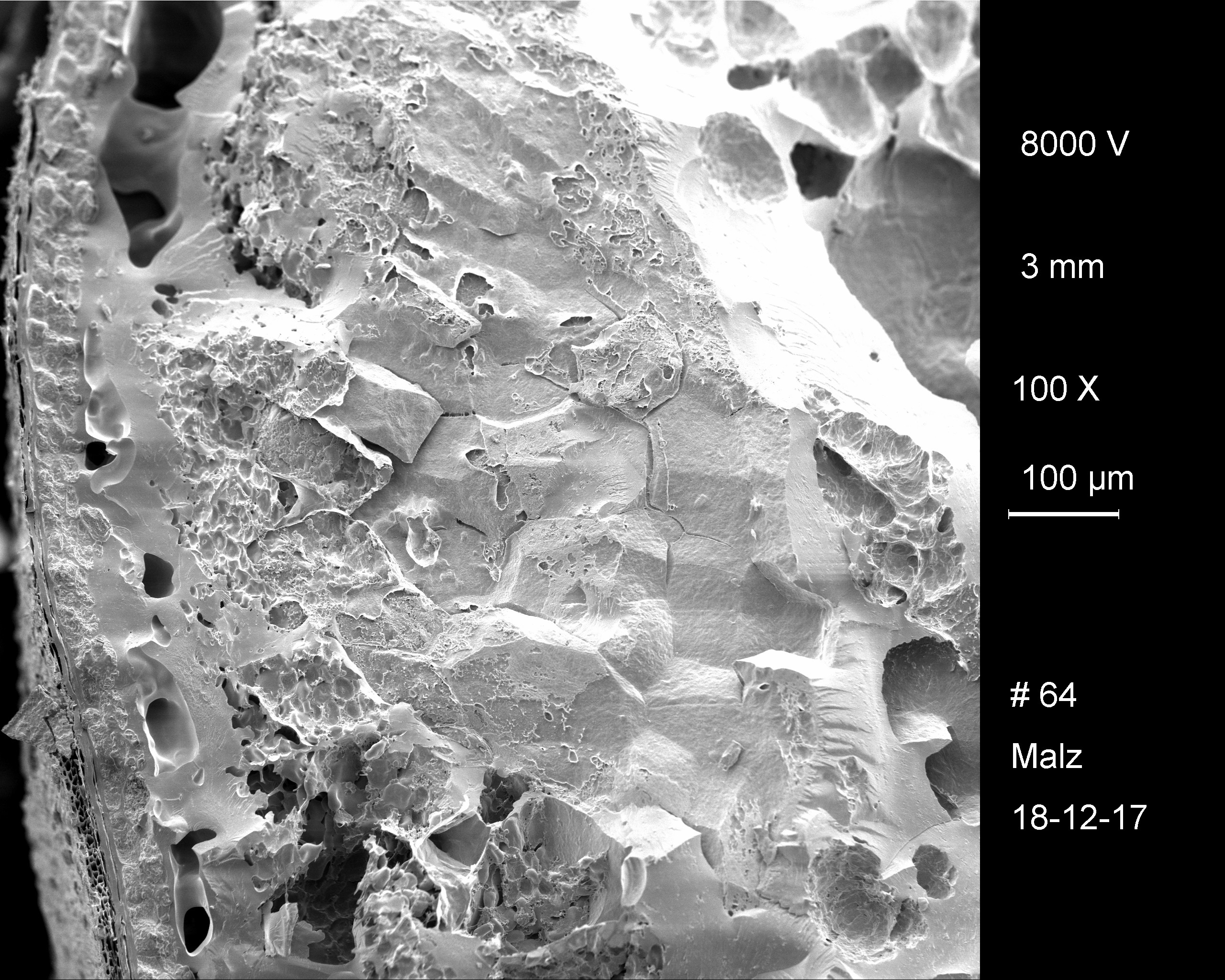

Supplement: S1 Archive — (ZIP) [file pone.0231696.s003.zip › HOVUS_M4_C_04.jpg]

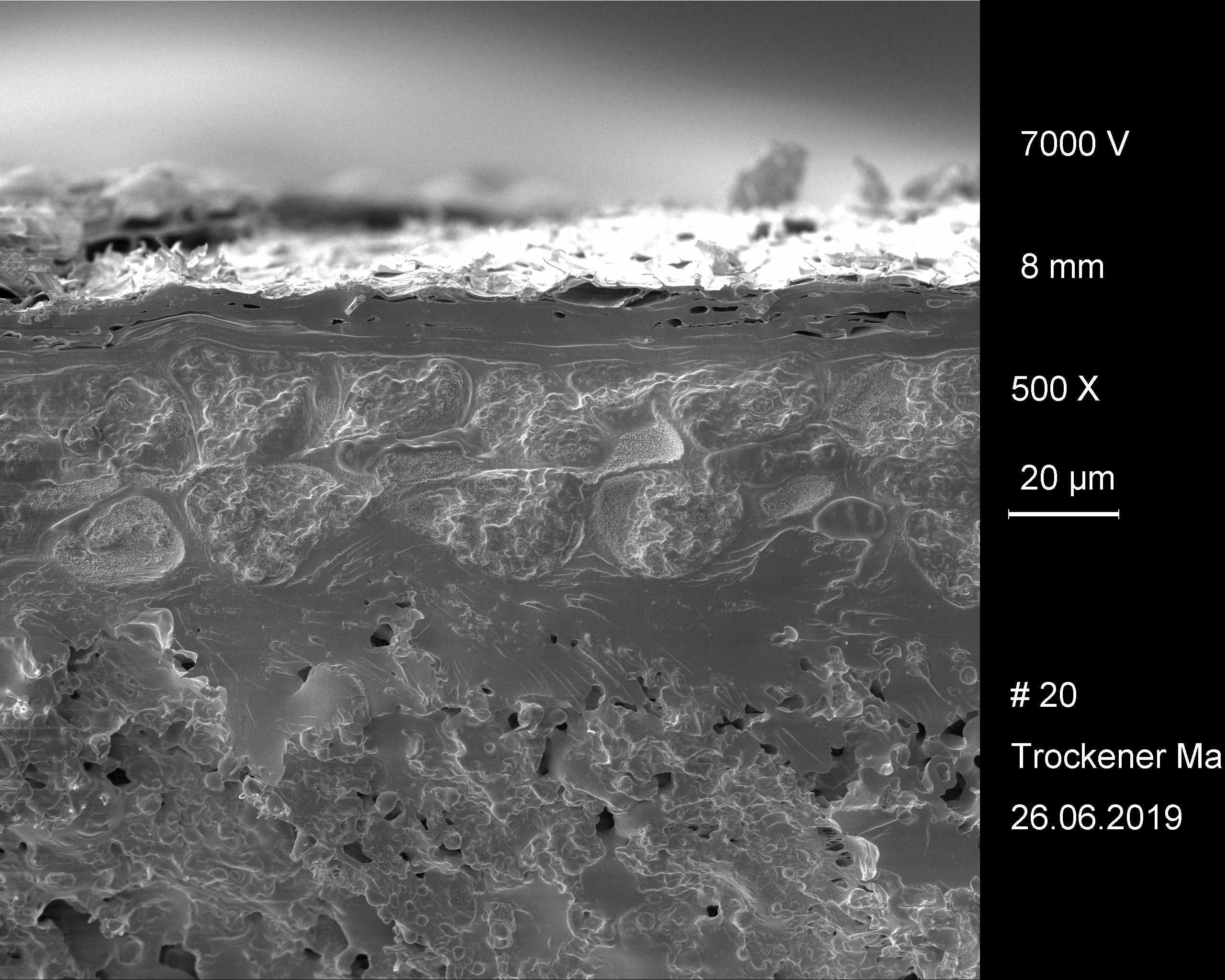

Supplement: S1 Archive — (ZIP) [file pone.0231696.s003.zip › HOVUS_M4_C_05.jpg]

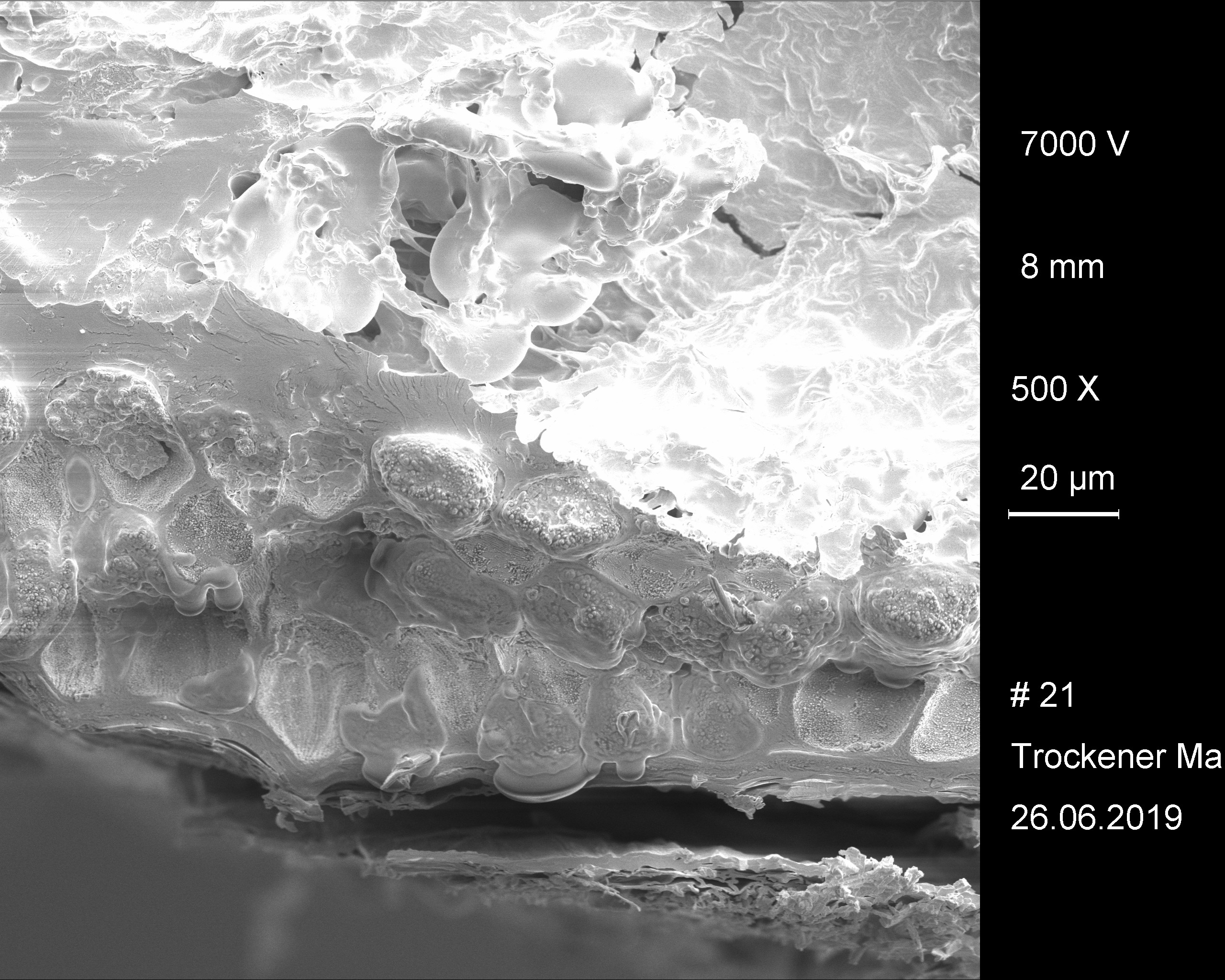

Supplement: S1 Archive — (ZIP) [file pone.0231696.s003.zip › HOVUS_M4_C_06.jpg]

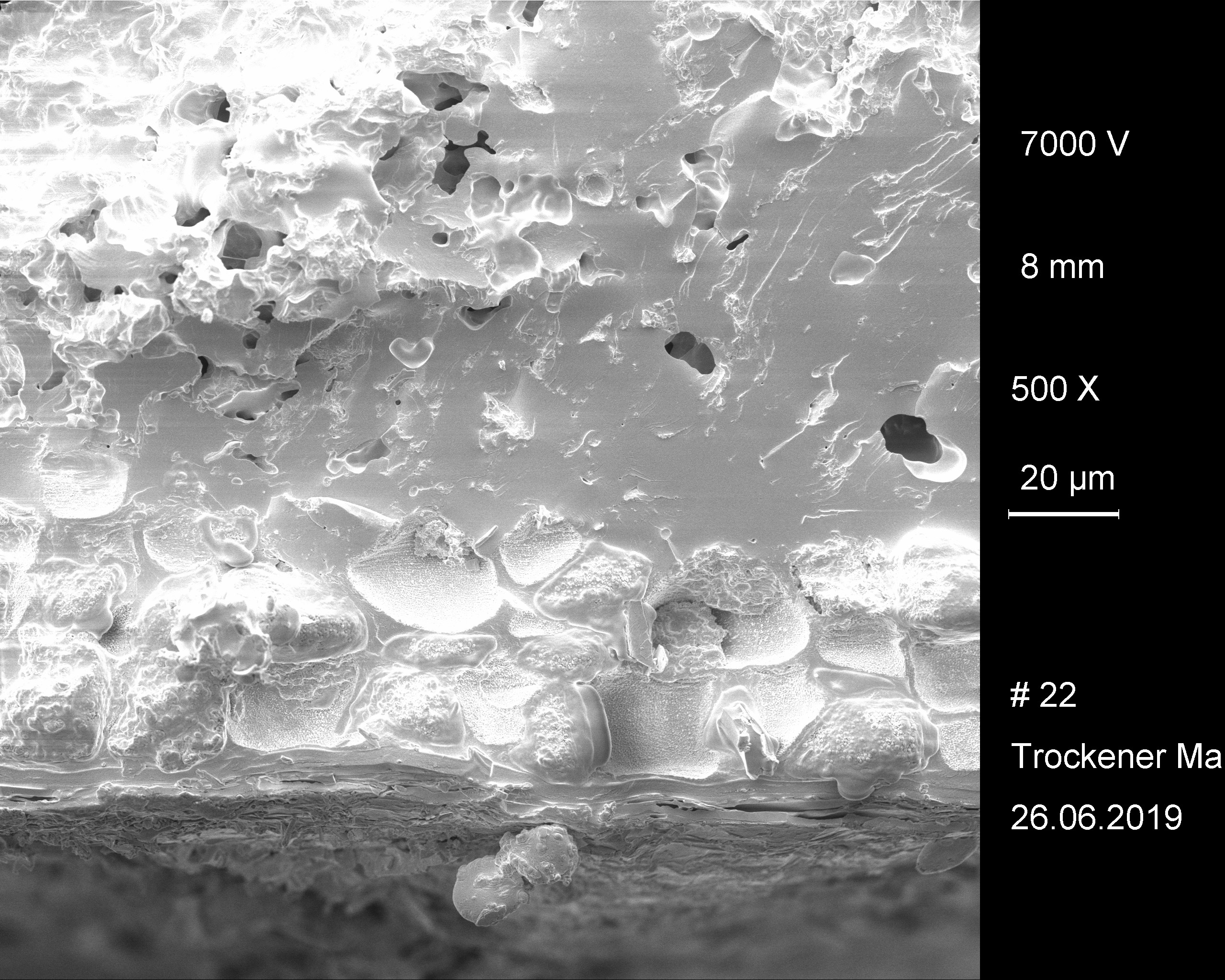

Supplement: S1 Archive — (ZIP) [file pone.0231696.s003.zip › HOVUS_M4_C_07.jpg]

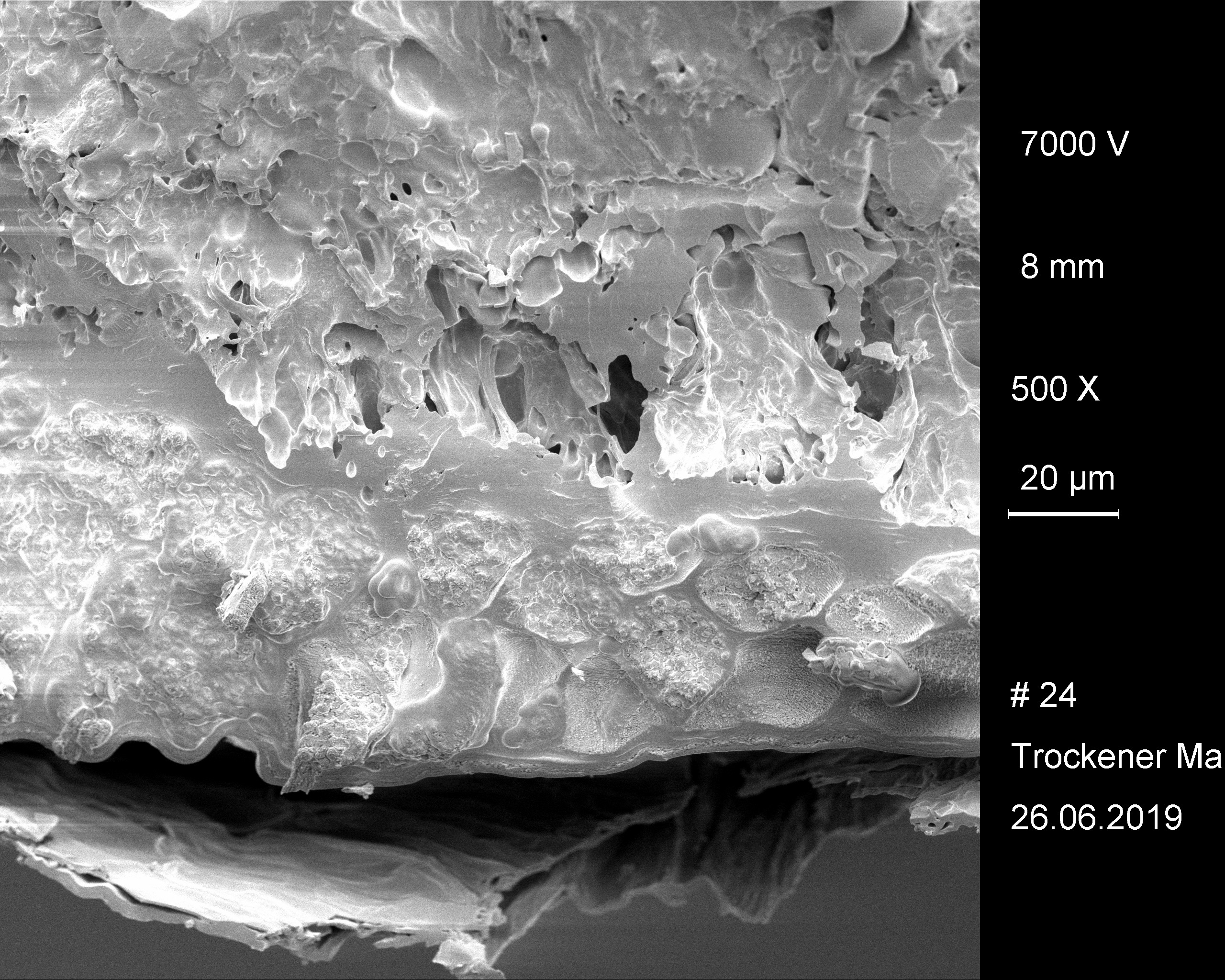

Supplement: S1 Archive — (ZIP) [file pone.0231696.s003.zip › HOVUS_M4_C_08.jpg]

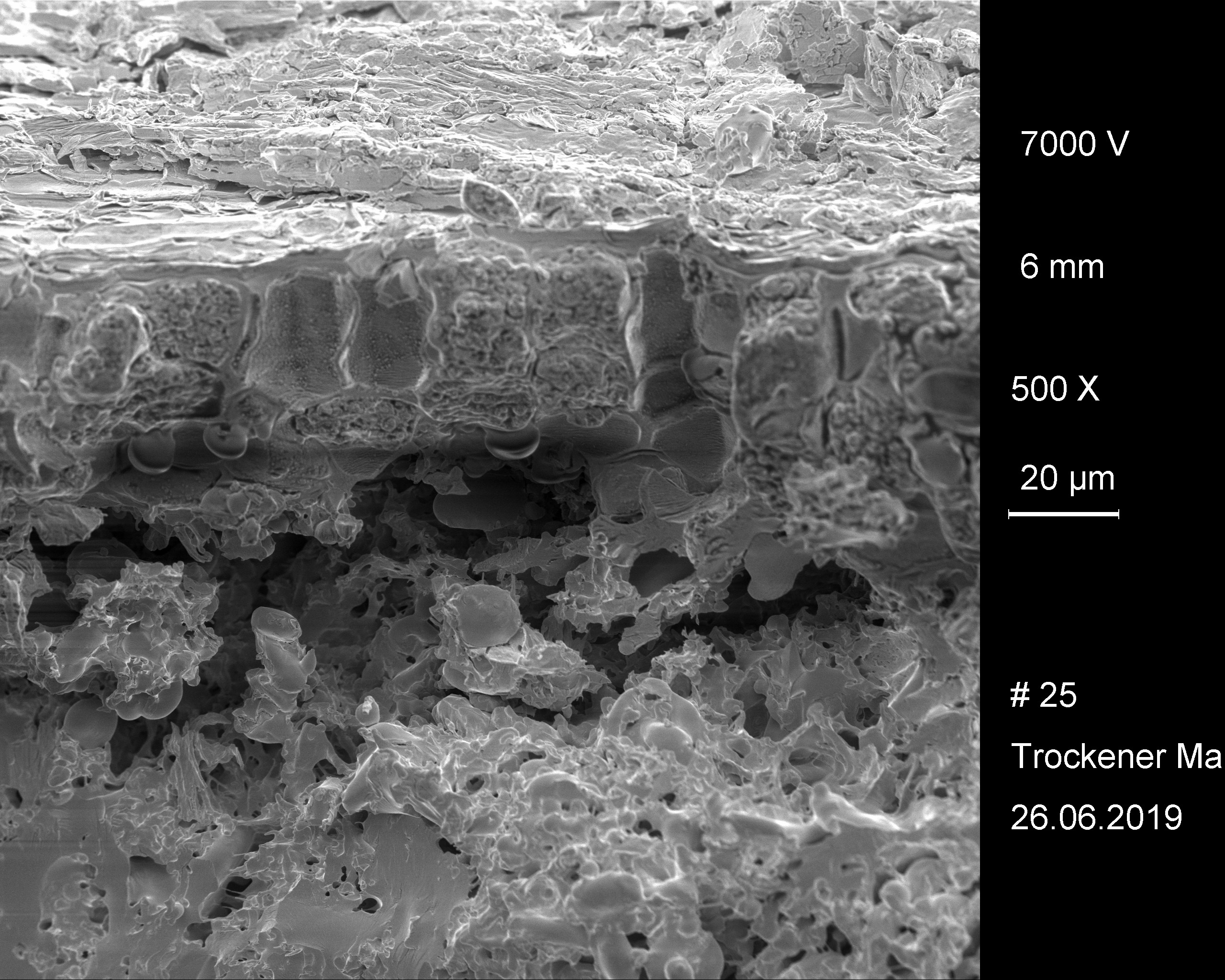

Supplement: S1 Archive — (ZIP) [file pone.0231696.s003.zip › HOVUS_M4_C_09.jpg]

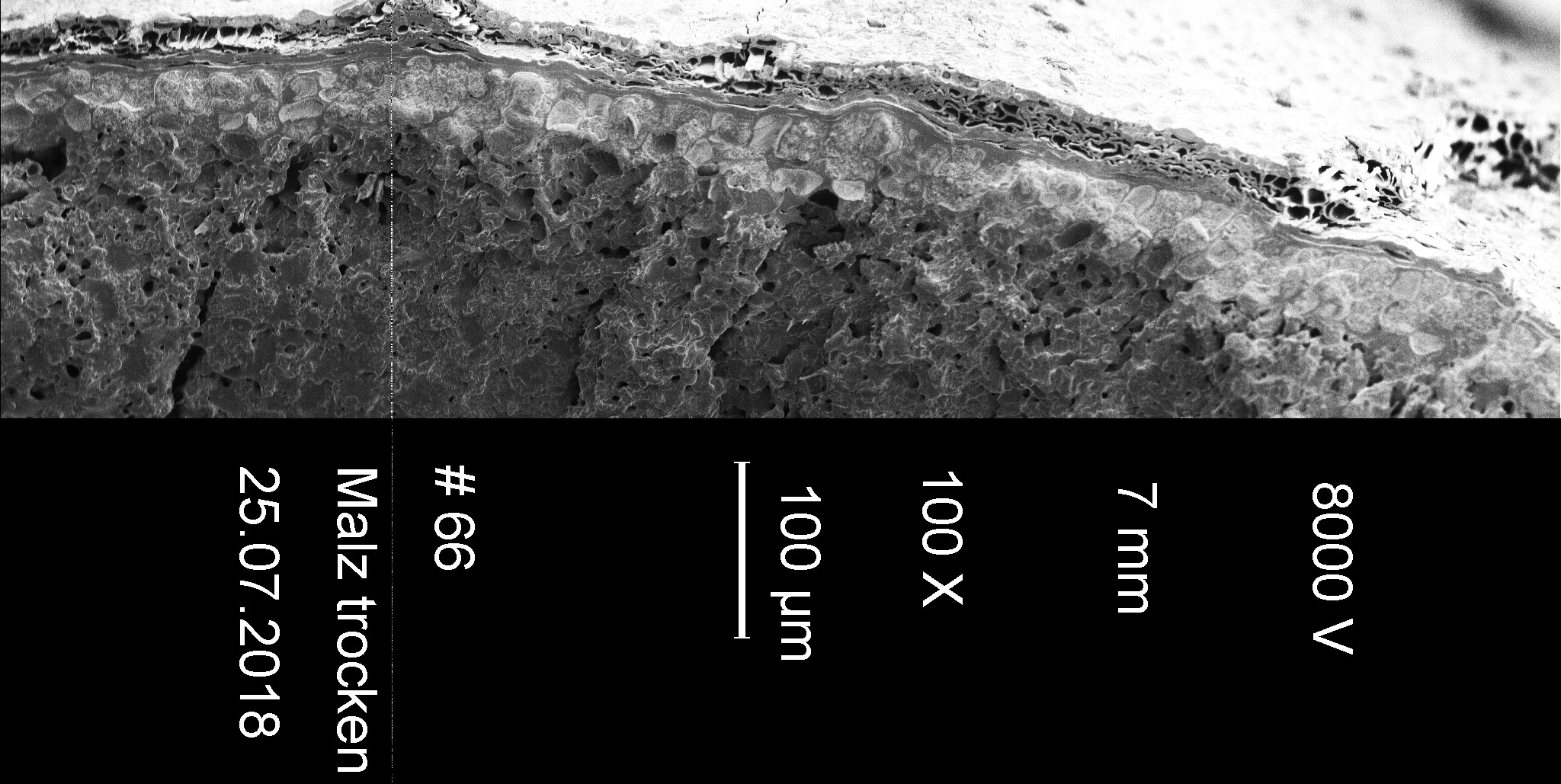

Supplement: S1 Archive — (ZIP) [file pone.0231696.s003.zip › HOVUS_M5_C_01.jpg]

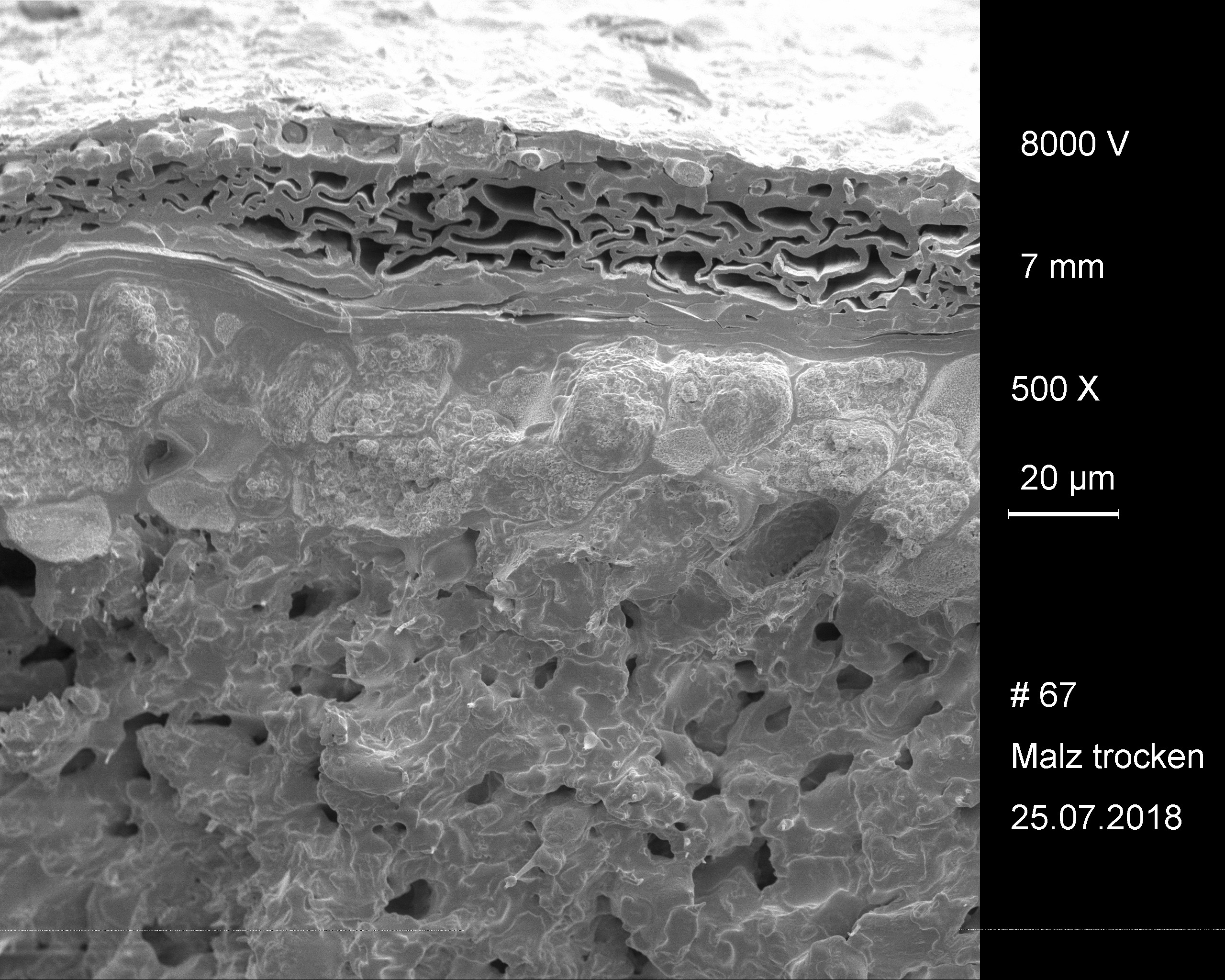

Supplement: S1 Archive — (ZIP) [file pone.0231696.s003.zip › HOVUS_M5_C_02.jpg]

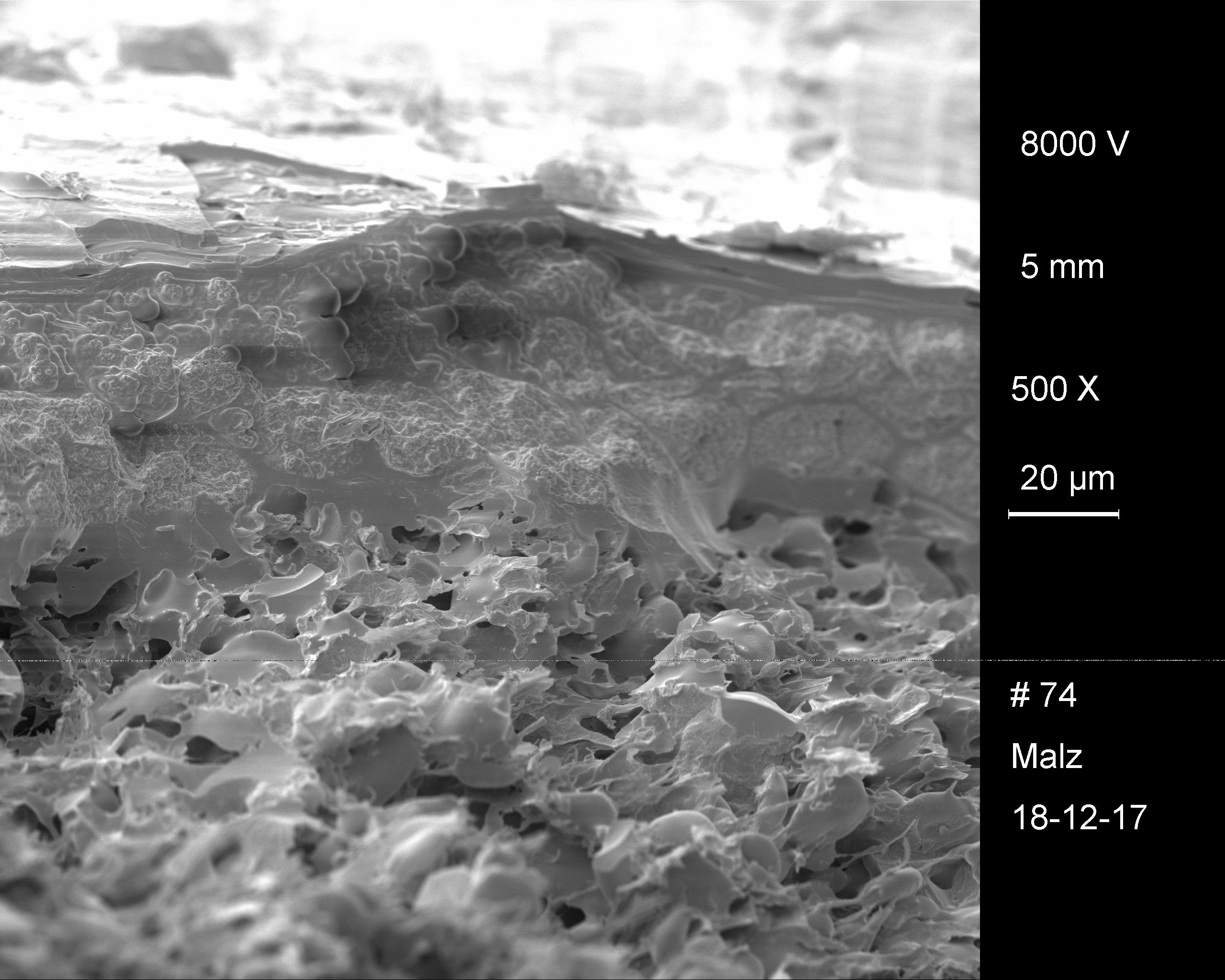

Supplement: S1 Archive — (ZIP) [file pone.0231696.s003.zip › HOVUS_M5_C_03.jpg]

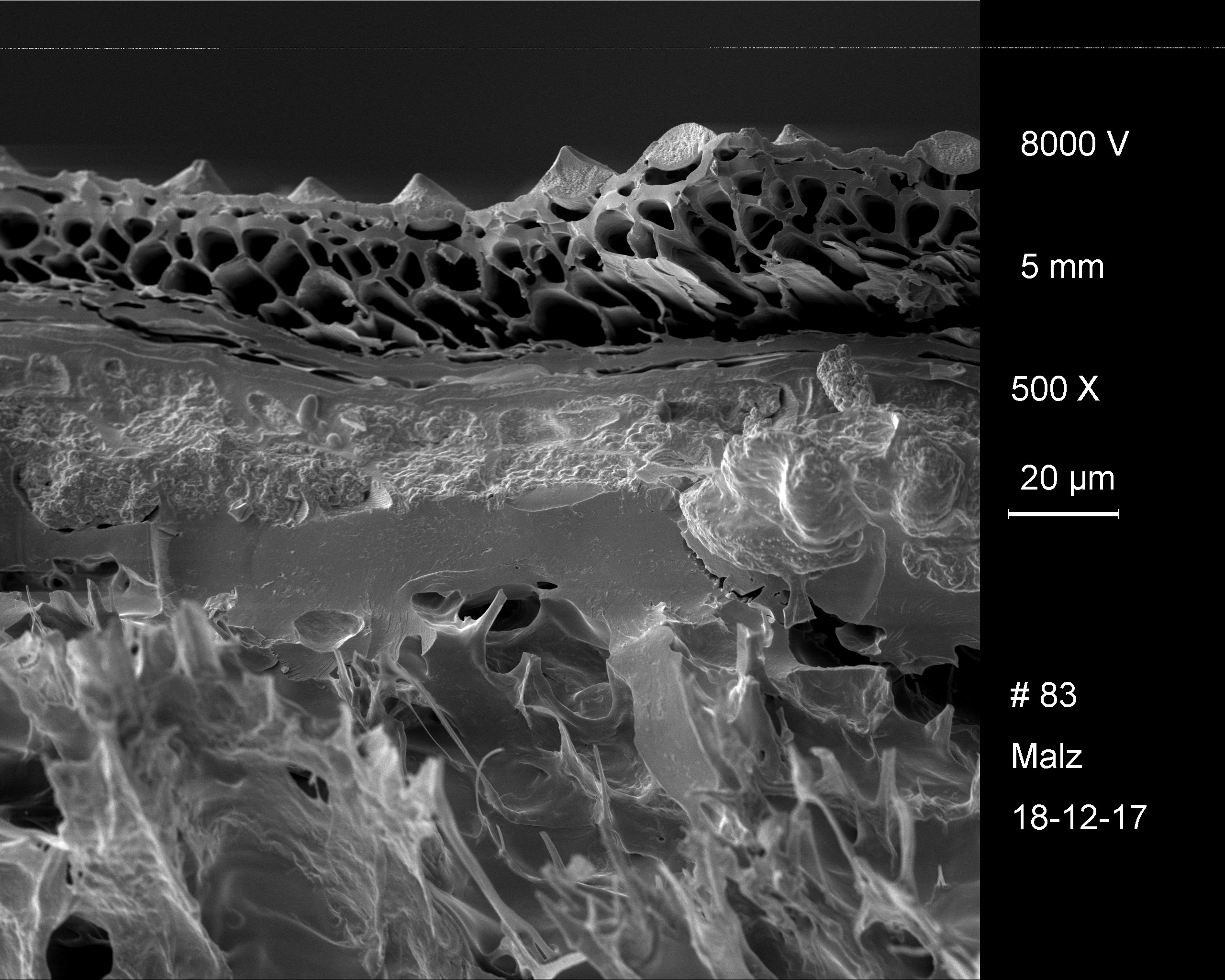

Supplement: S1 Archive — (ZIP) [file pone.0231696.s003.zip › HOVUS_M5_C_04.jpg]

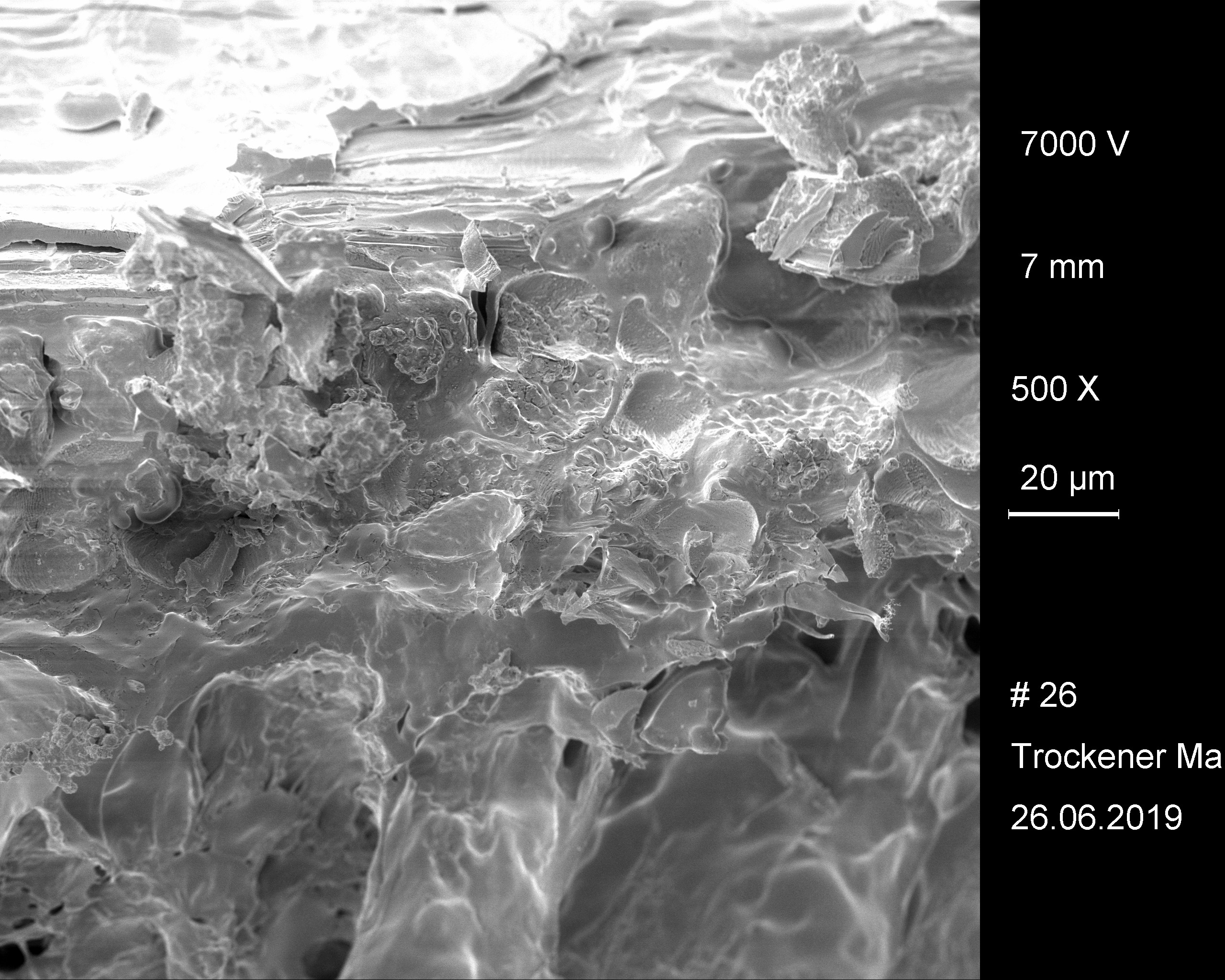

Supplement: S1 Archive — (ZIP) [file pone.0231696.s003.zip › HOVUS_M5_C_05.jpg]

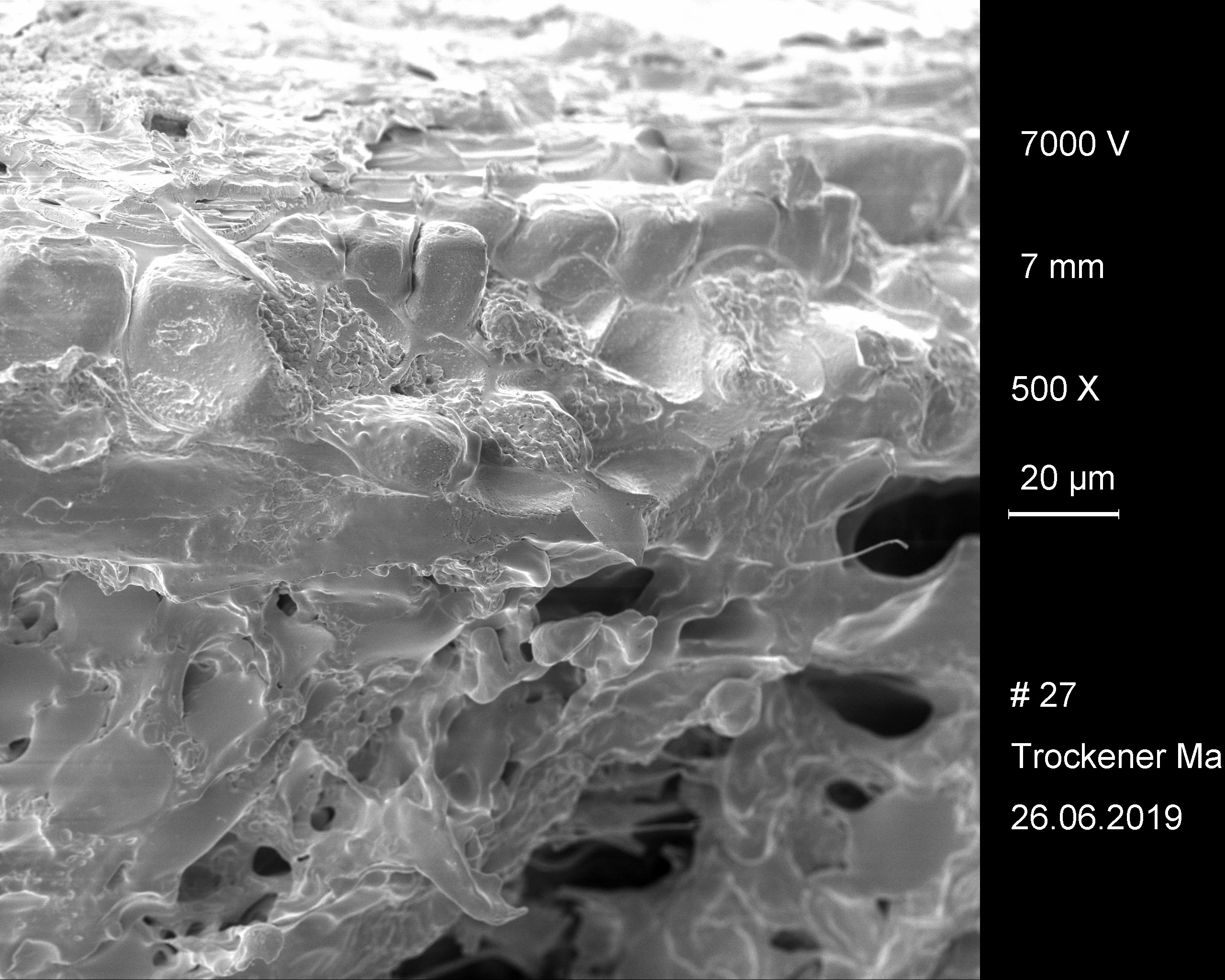

Supplement: S1 Archive — (ZIP) [file pone.0231696.s003.zip › HOVUS_M5_C_06.jpg]

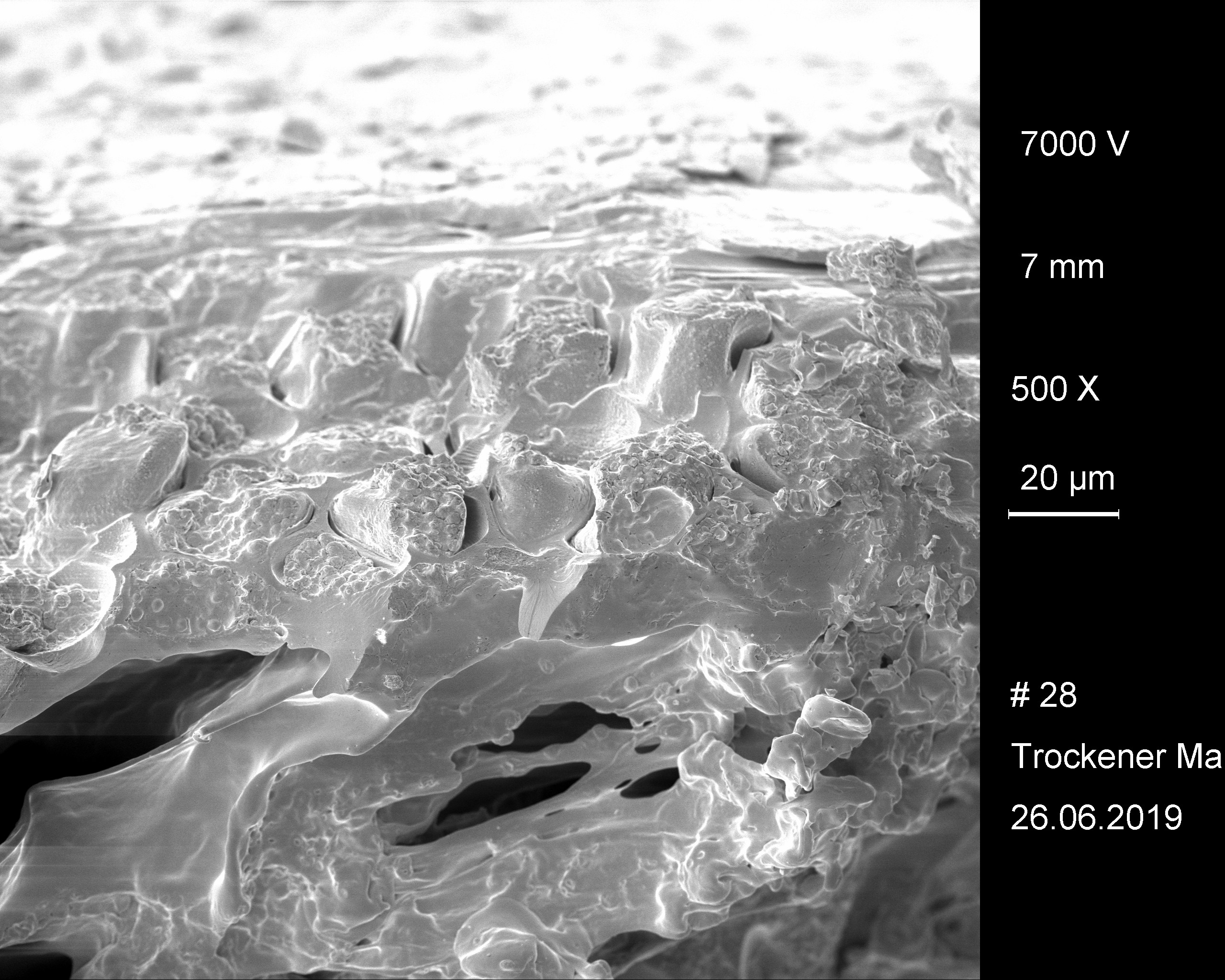

Supplement: S1 Archive — (ZIP) [file pone.0231696.s003.zip › HOVUS_M5_C_07.jpg]

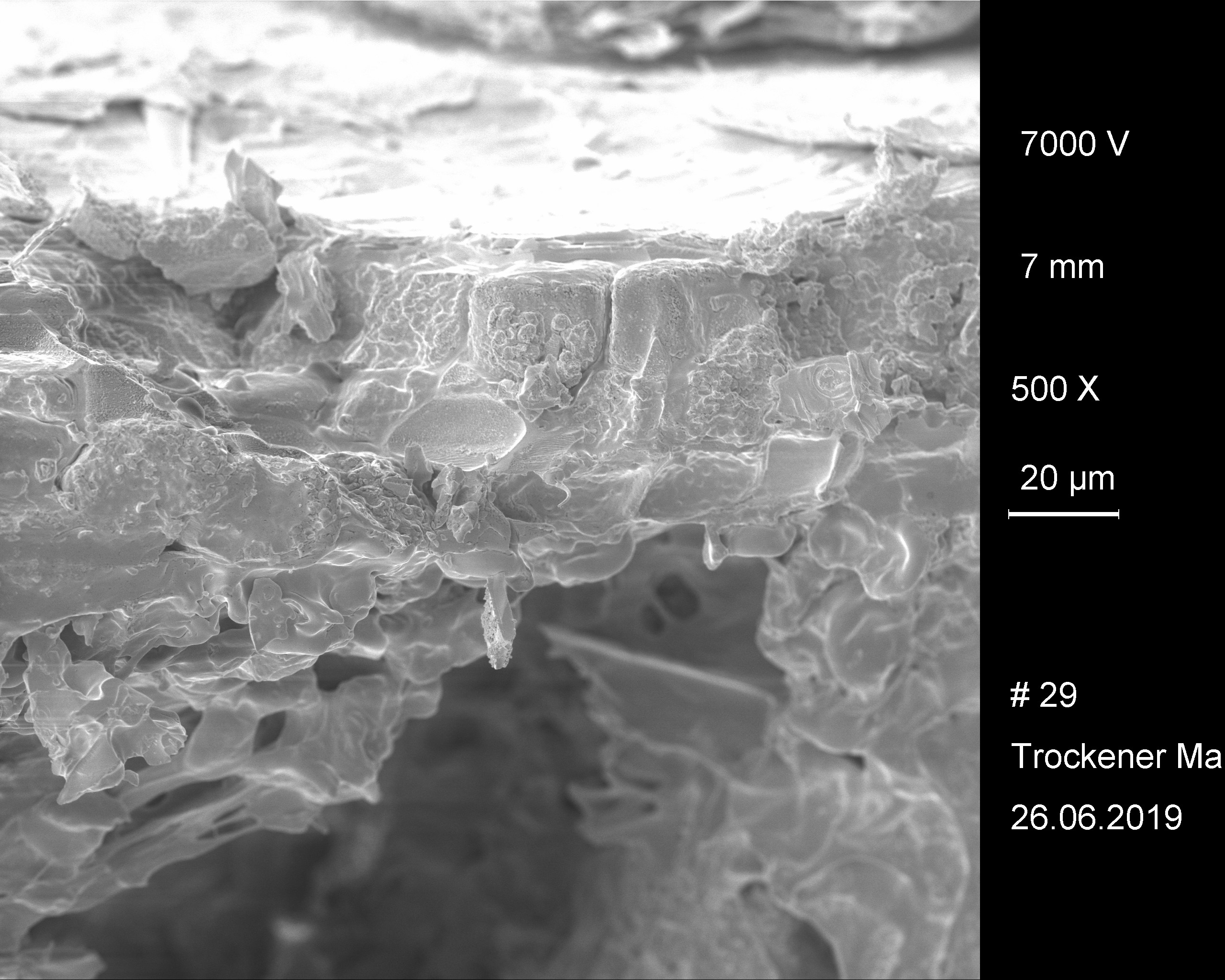

Supplement: S1 Archive — (ZIP) [file pone.0231696.s003.zip › HOVUS_M5_C_08.jpg]

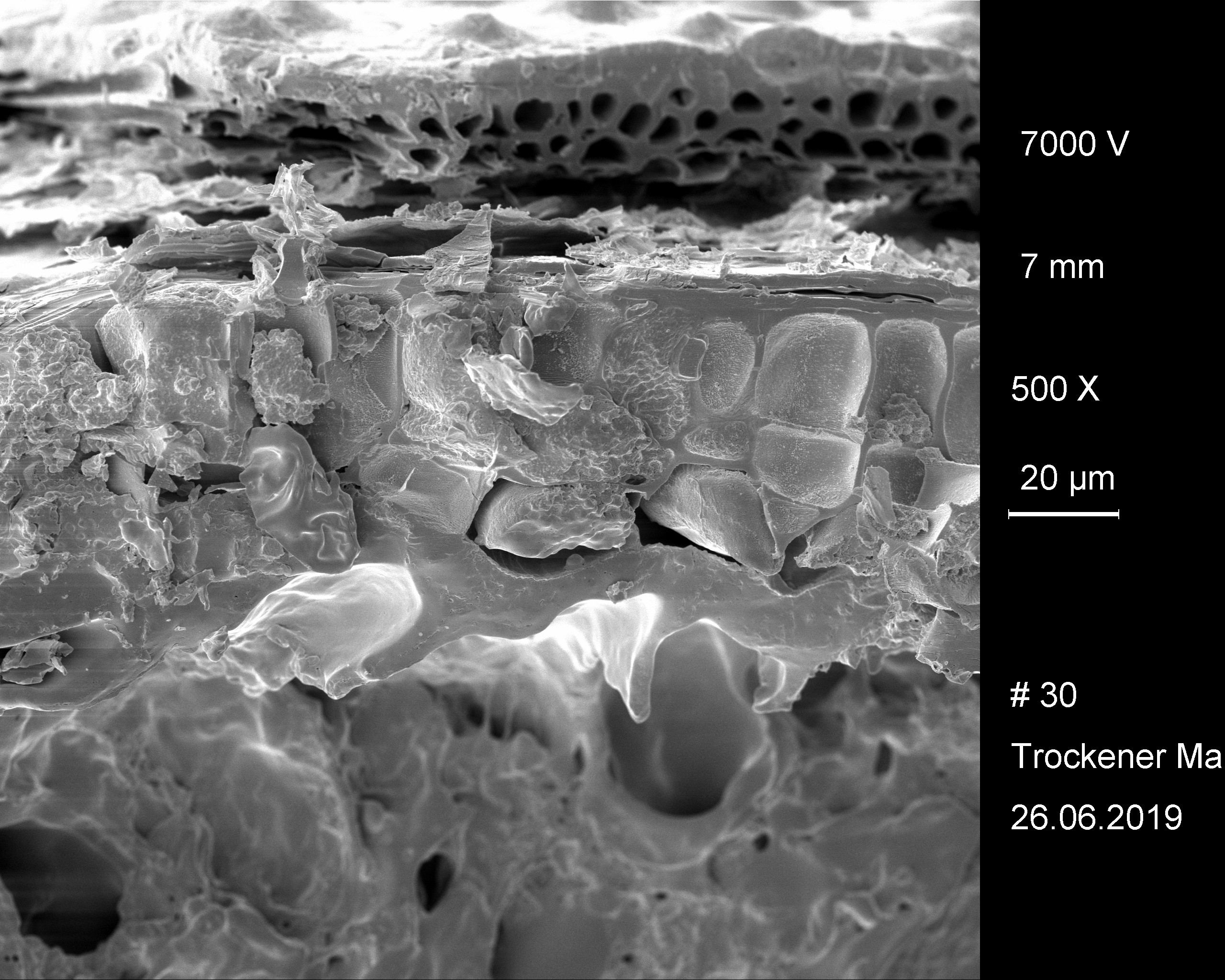

Supplement: S1 Archive — (ZIP) [file pone.0231696.s003.zip › HOVUS_M5_C_09.jpg]

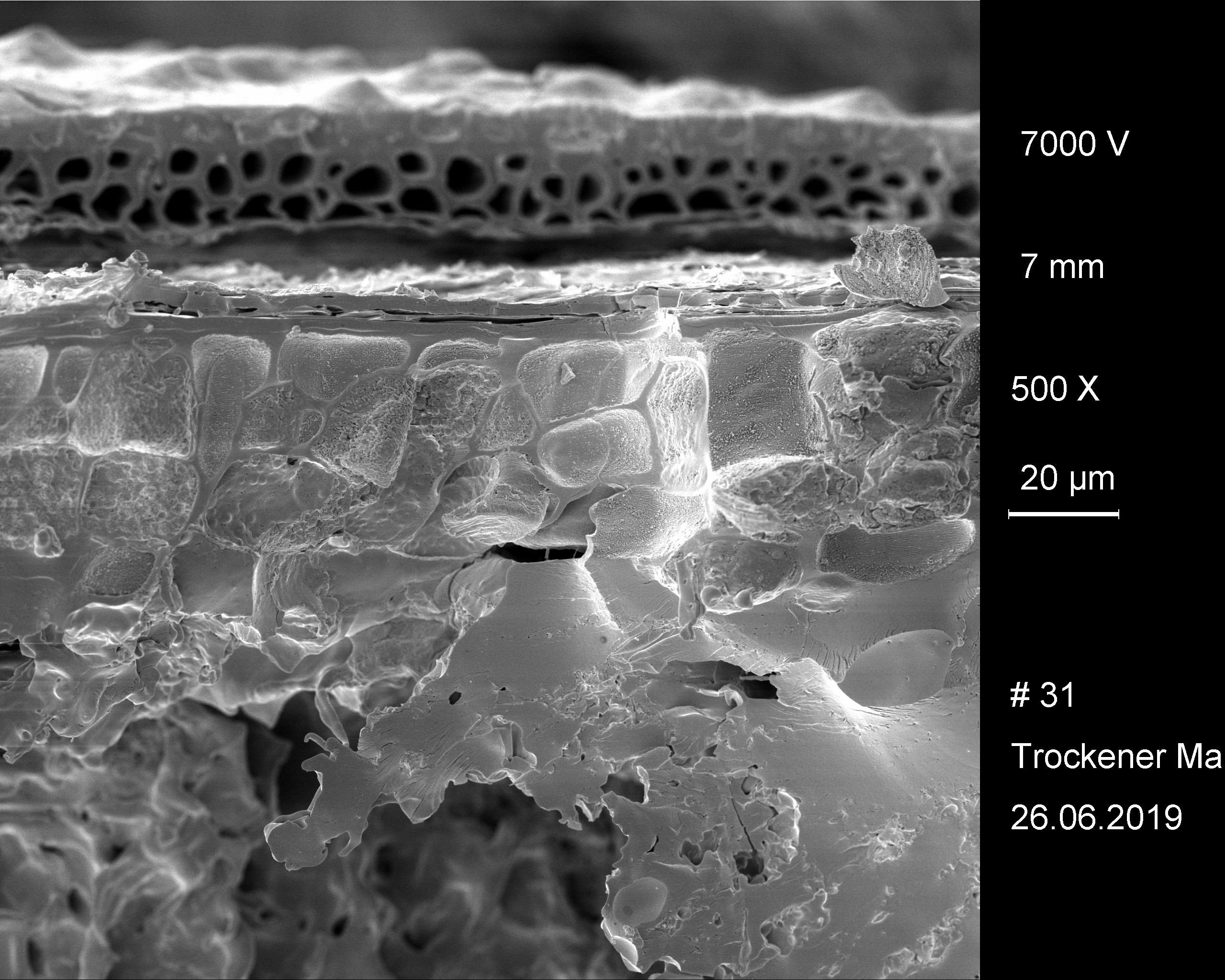

Supplement: S1 Archive — (ZIP) [file pone.0231696.s003.zip › HOVUS_M5_C_10.jpg]

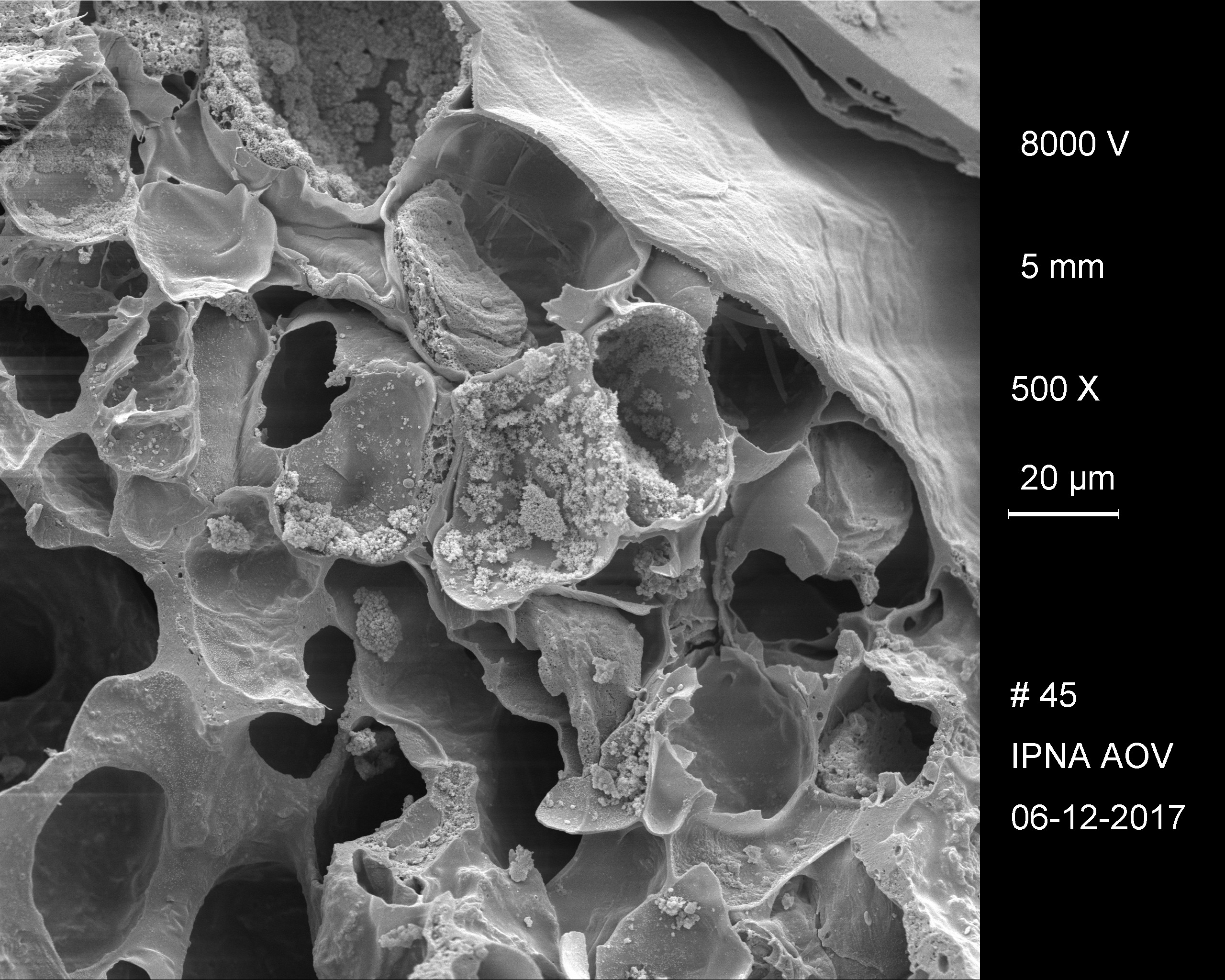

Supplement: S2 Archive — (ZIP) [file pone.0231696.s004.zip › AOV_85 45.jpg]

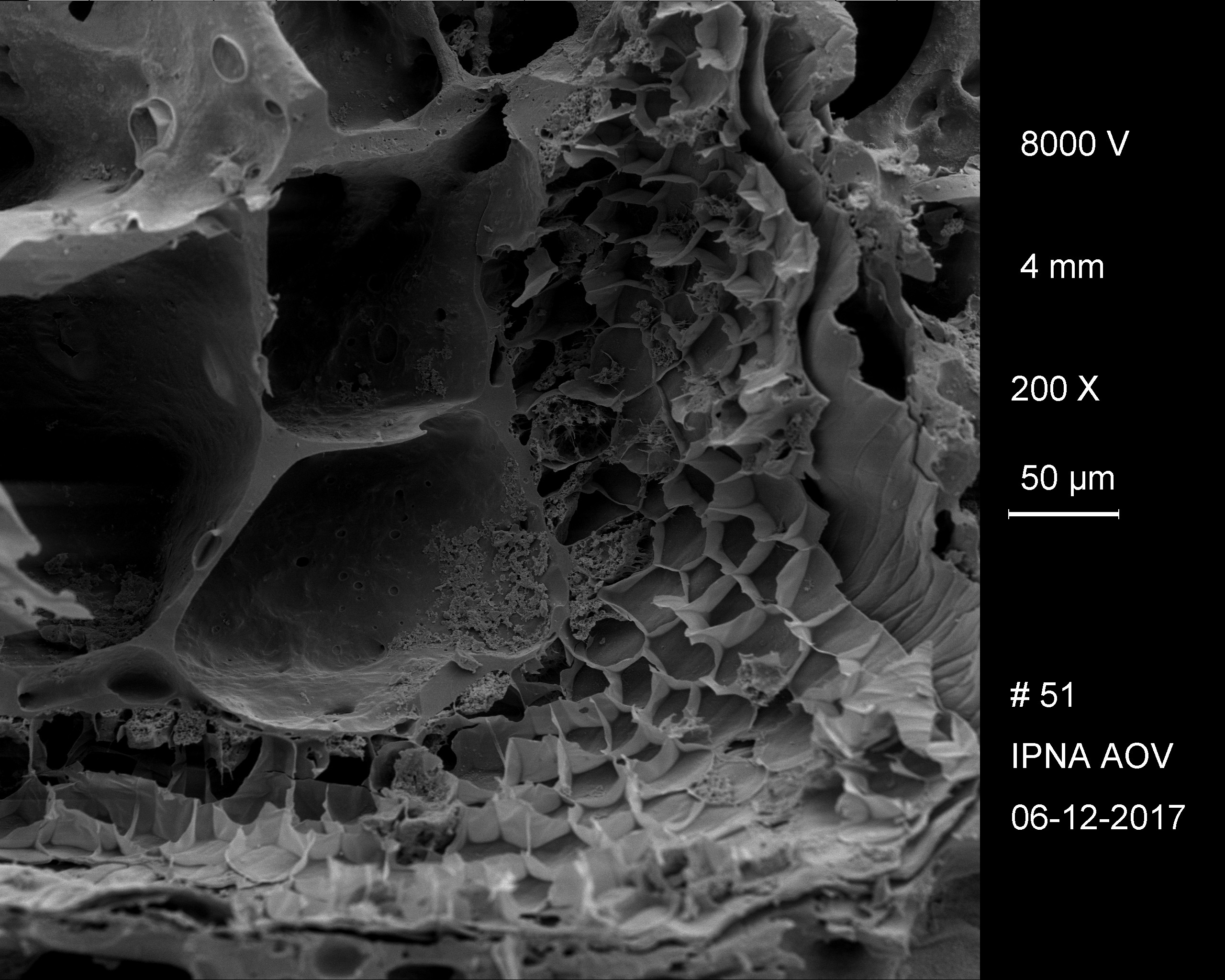

Supplement: S2 Archive — (ZIP) [file pone.0231696.s004.zip › AOV_85 51.jpg]

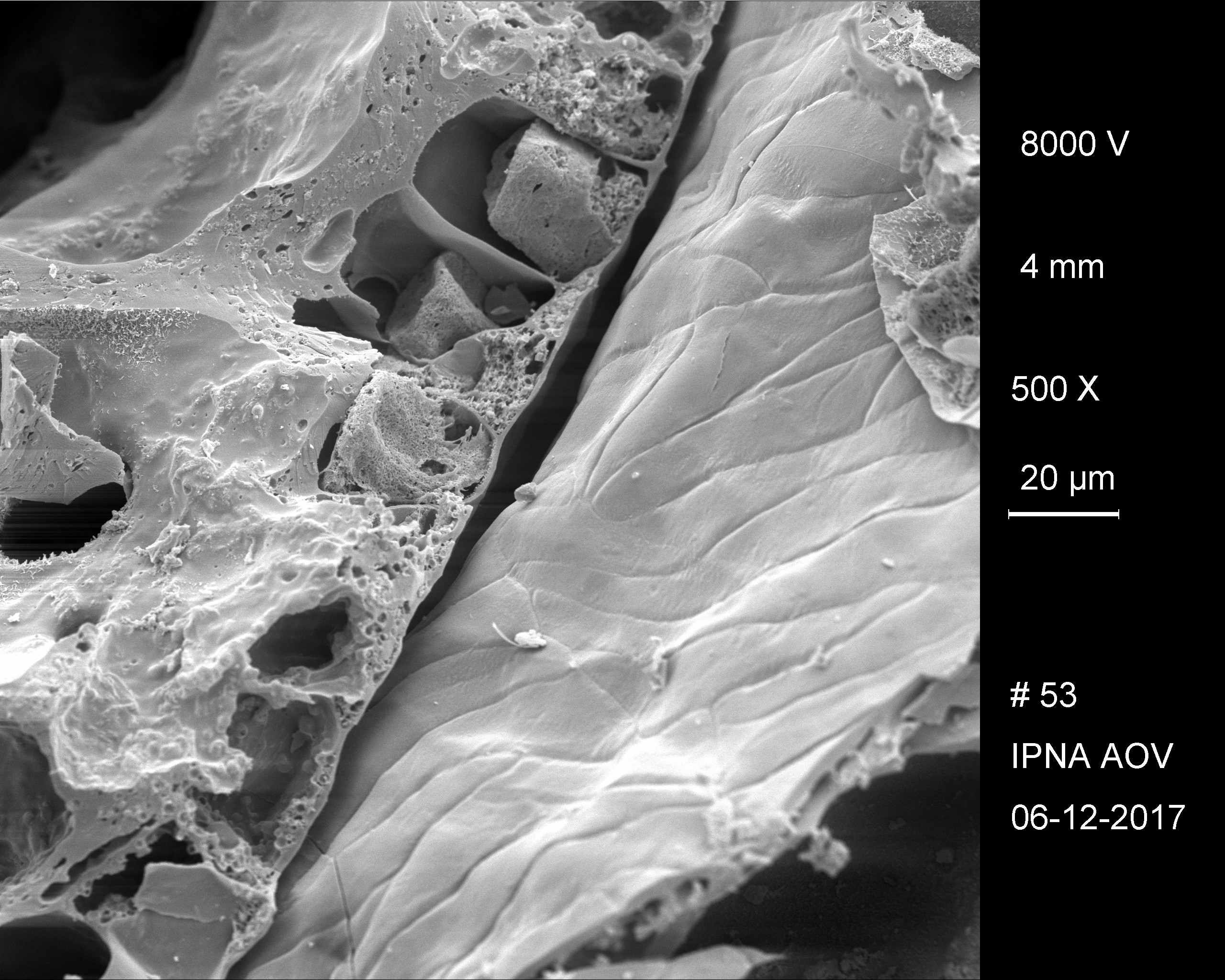

Supplement: S2 Archive — (ZIP) [file pone.0231696.s004.zip › AOV_85 53.jpg]

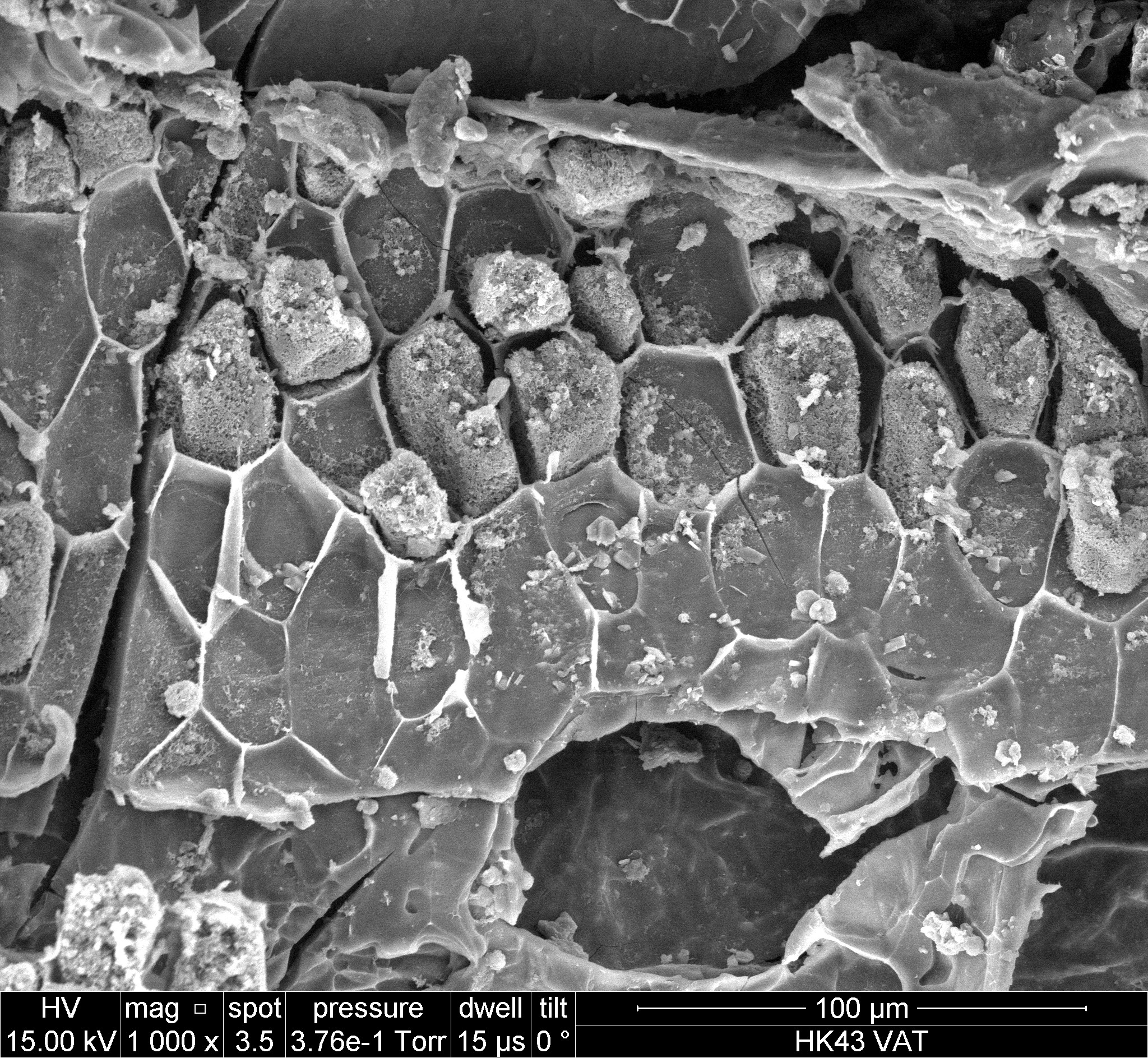

Supplement: S2 Archive — (ZIP) [file pone.0231696.s004.zip › HK11C VAT-06.jpg]

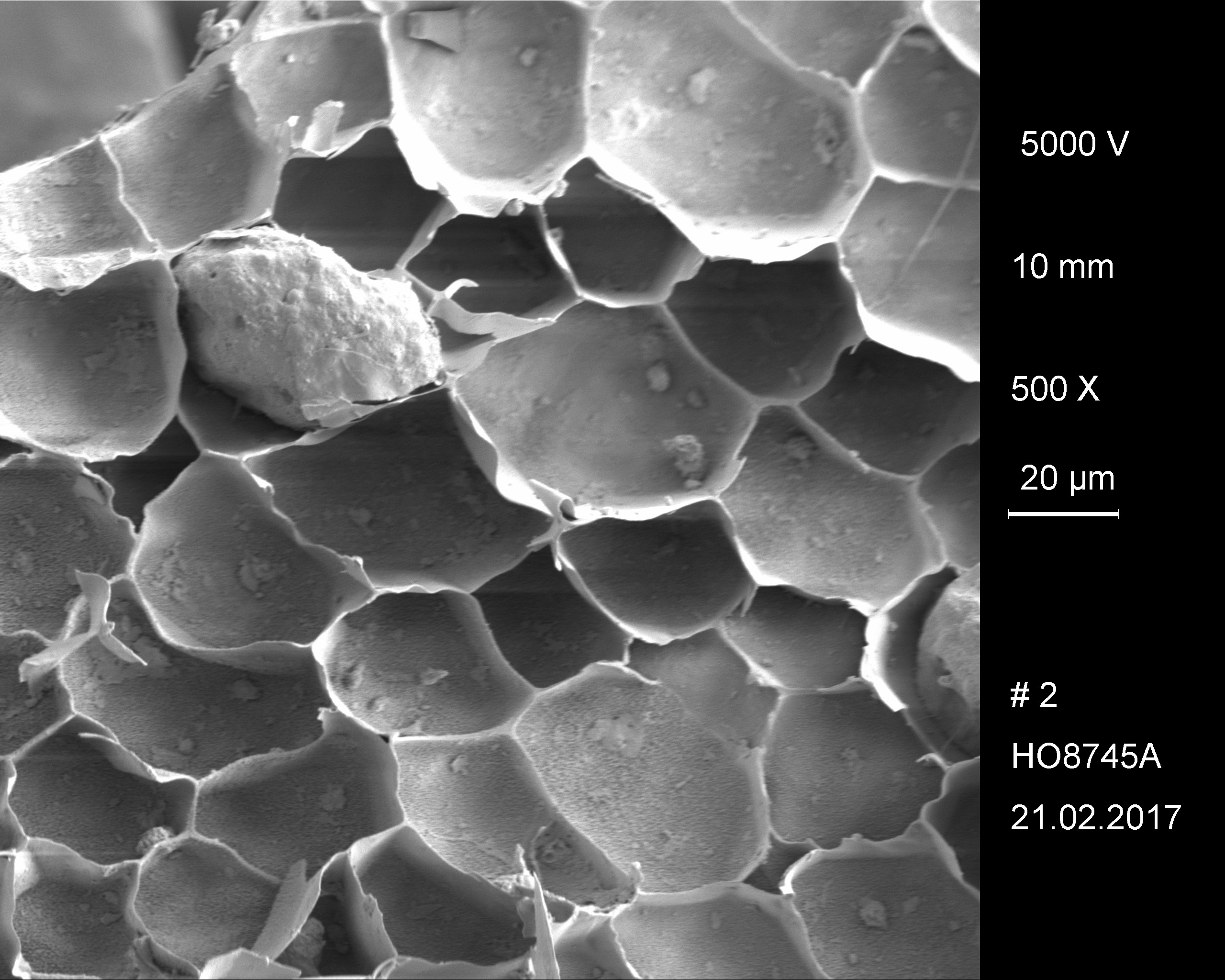

Supplement: S2 Archive — (ZIP) [file pone.0231696.s004.zip › HO8745A_2.jpg]

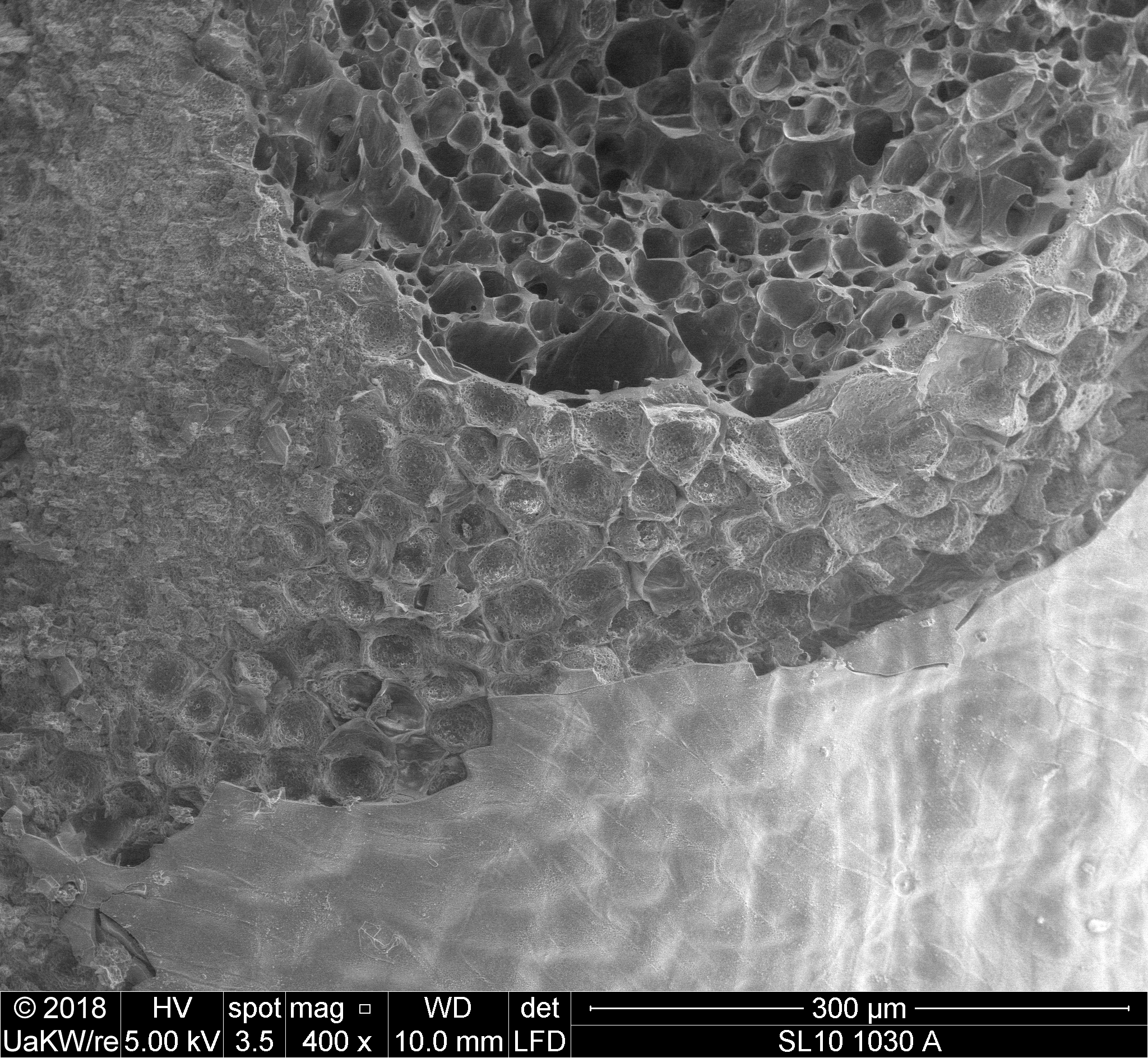

Supplement: S2 Archive — (ZIP) [file pone.0231696.s004.zip › SL10_1030_A_03.tif]

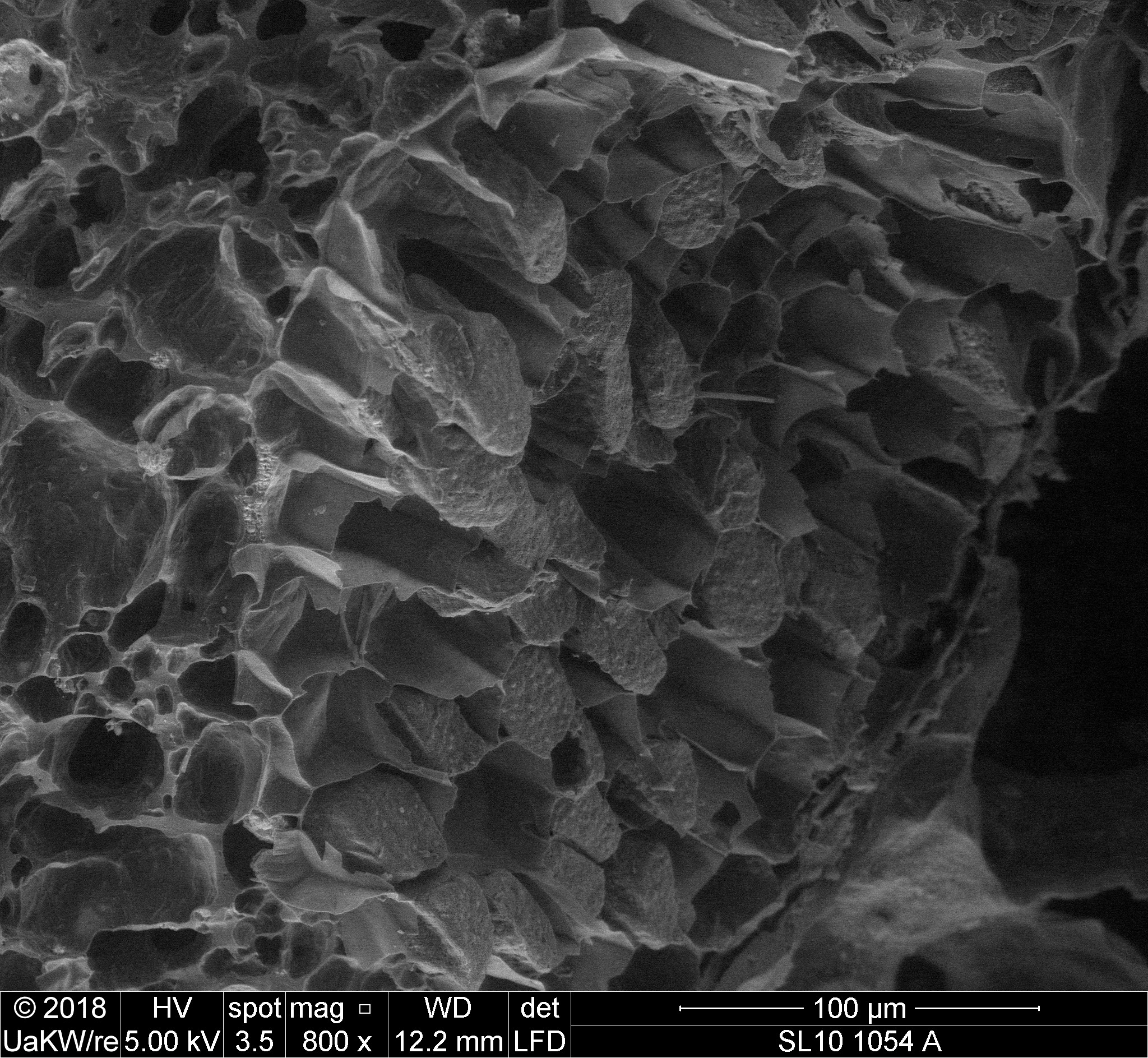

Supplement: S2 Archive — (ZIP) [file pone.0231696.s004.zip › SL10_1054_A_04.jpg]

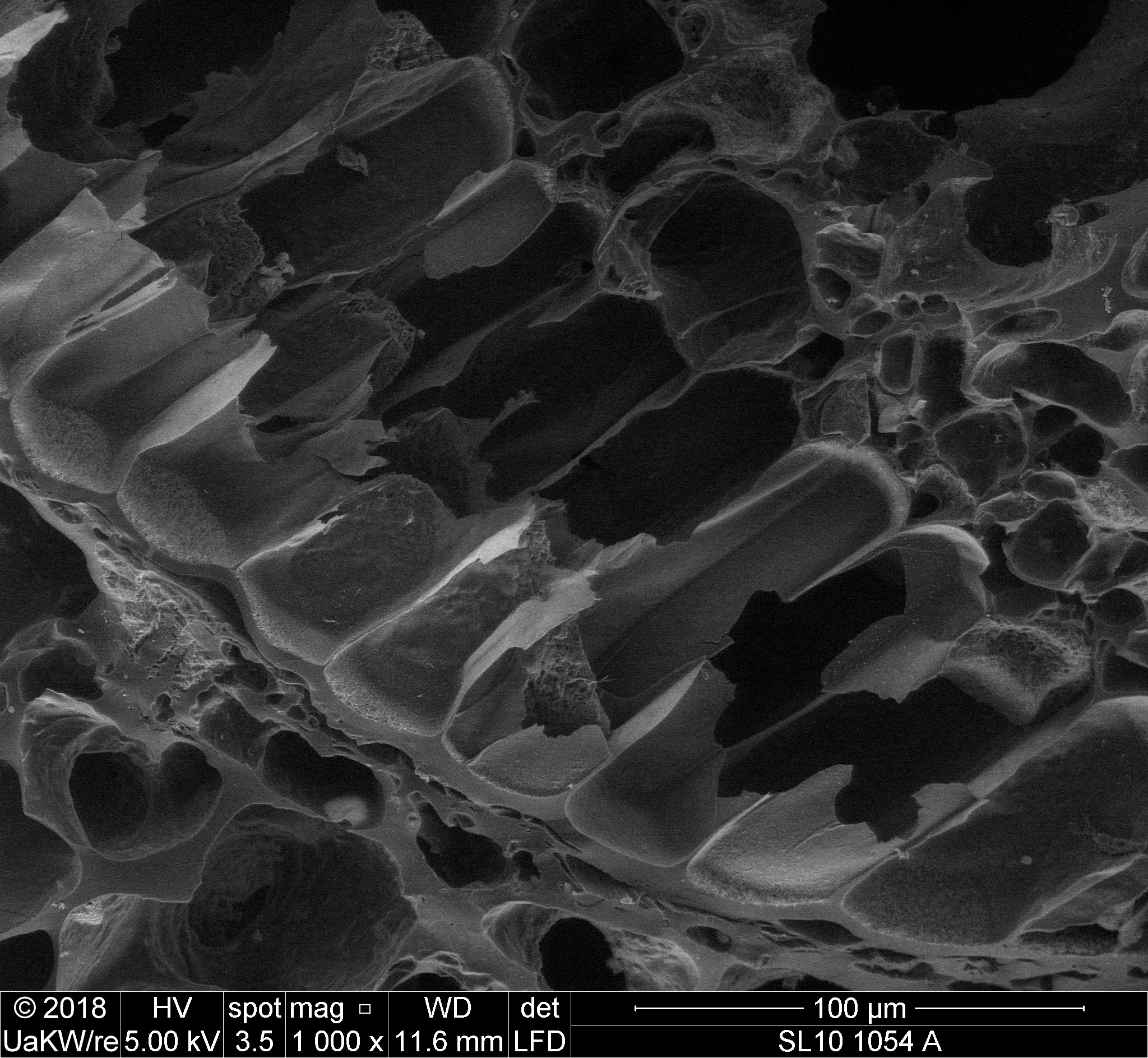

Supplement: S2 Archive — (ZIP) [file pone.0231696.s004.zip › SL10_1054_A_07.jpg]

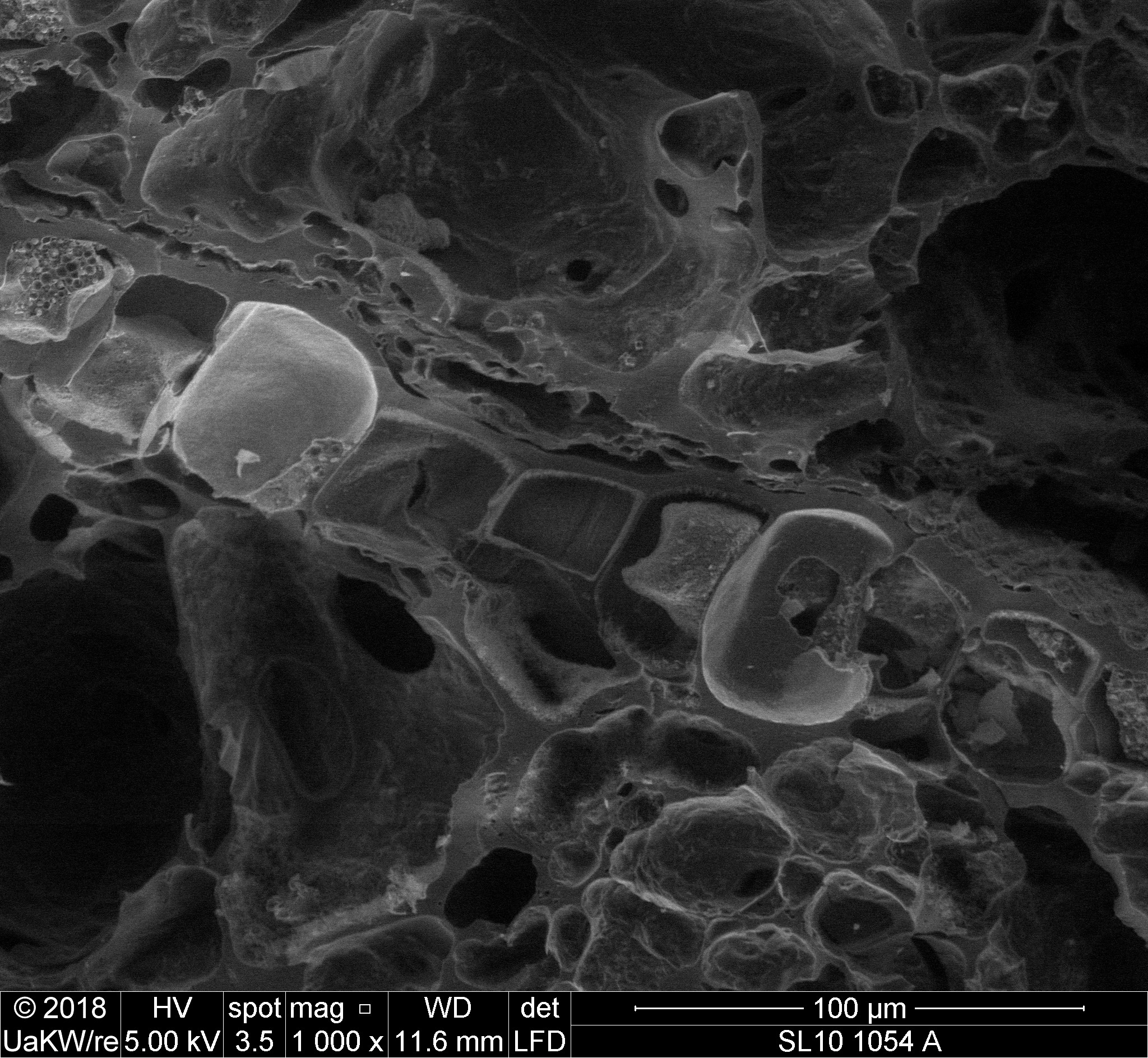

Supplement: S2 Archive — (ZIP) [file pone.0231696.s004.zip › SL10_1054_A_08.jpg]

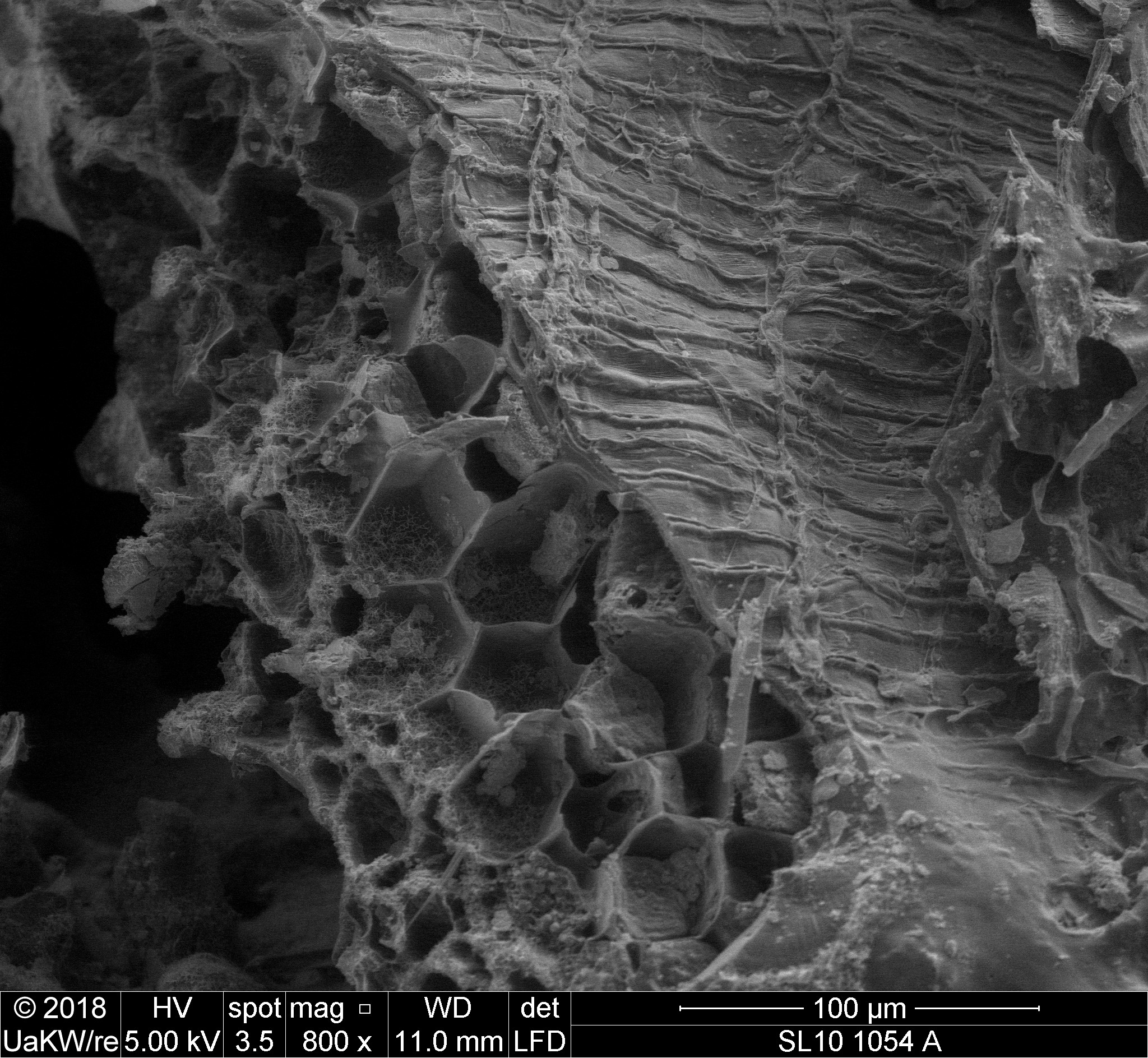

Supplement: S2 Archive — (ZIP) [file pone.0231696.s004.zip › SL10_1054_A_10.jpg]

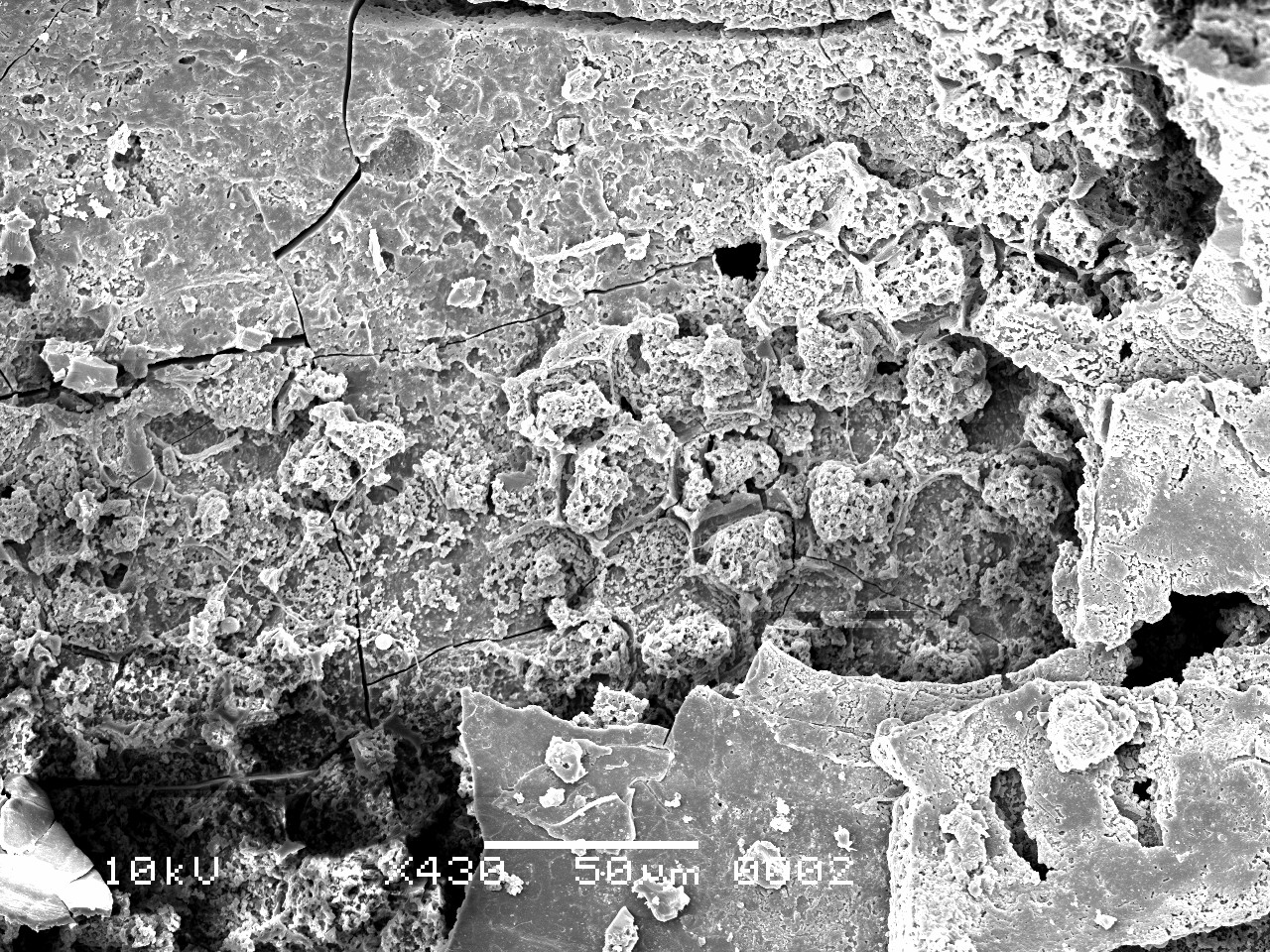

Supplement: S2 Archive — (ZIP) [file pone.0231696.s004.zip › Tell el-Farkha feature 200 stub 1 foto 02.jpg]

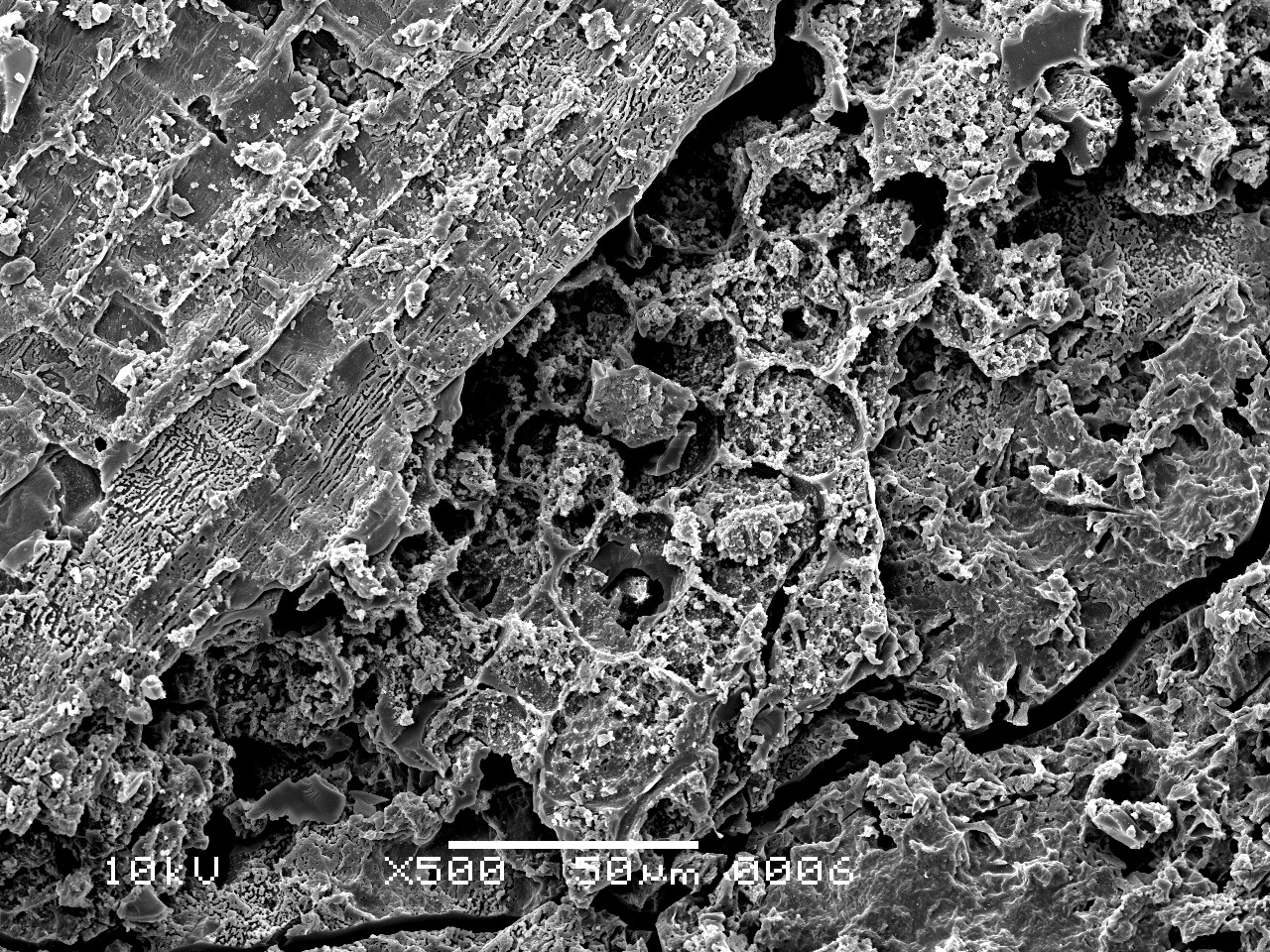

Supplement: S2 Archive — (ZIP) [file pone.0231696.s004.zip › Tell el-Farkha feature 200 stub 4 foto 06.jpg]

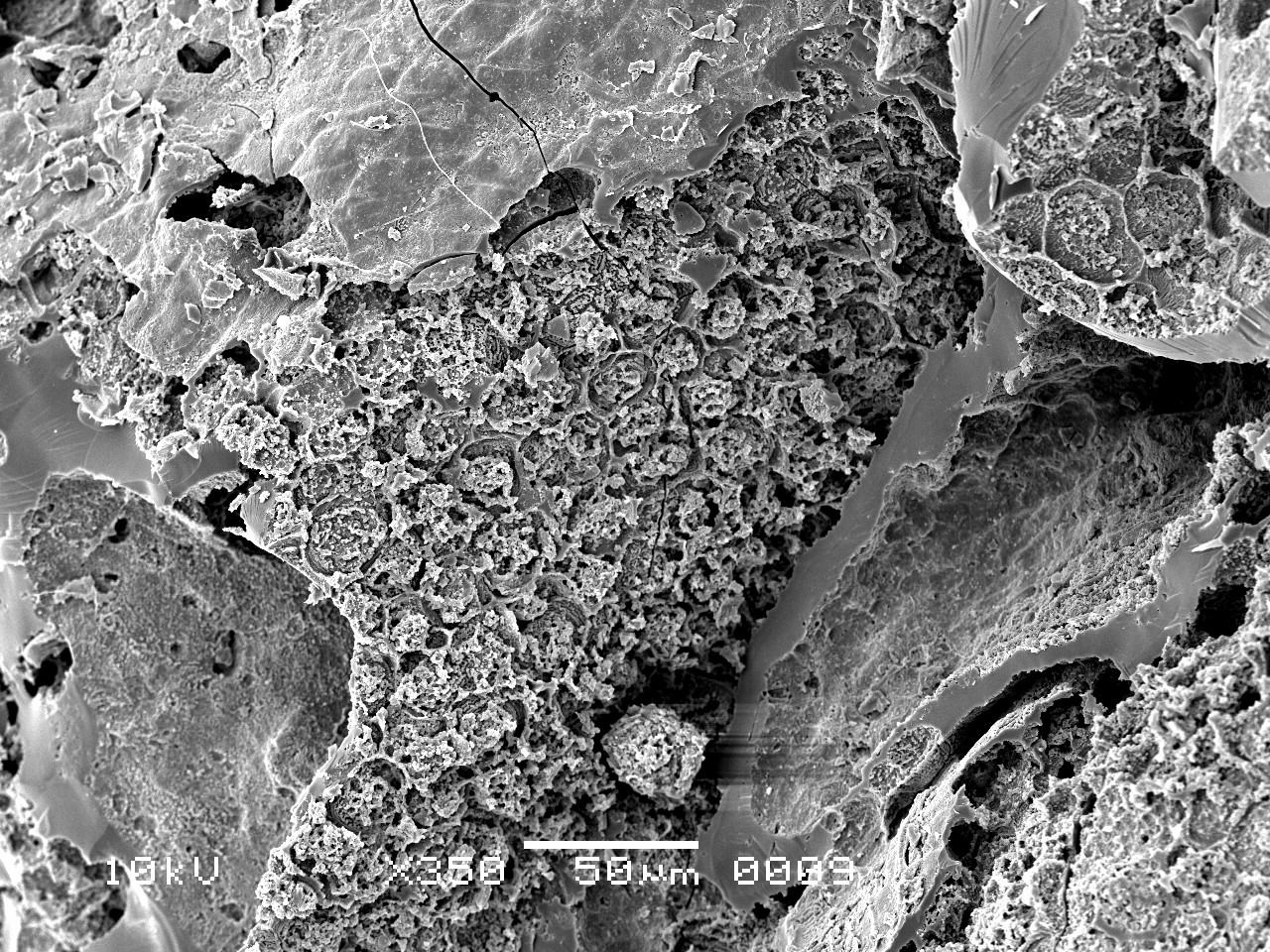

Supplement: S2 Archive — (ZIP) [file pone.0231696.s004.zip › Tell el-Farkha feature 200 stub 4 foto 09.jpg]
